# Structural refinement and electrochemical properties of one dimensional (ZnO NRs)_1−x_(CNs)_x_ functional hybrids for serotonin sensing studies

**DOI:** 10.1038/s41598-020-72756-3

**Published:** 2020-09-29

**Authors:** Sajid B. Mullani, Ananta G. Dhodamani, Annadanesh Shellikeri, Navaj B. Mullani, Anita K. Tawade, Shivaji N. Tayade, Julien Biscay, Lynn Dennany, Sagar D. Delekar

**Affiliations:** 1 Department of Chemistry, Shivaji University, Kolhapur, MS 416004 India; 2 Department of Electrical and Computer Engineering, Florida A&M University-Florida State University, Tallahassee, FL 32310-6046 USA; 3 Aero-Propulsion, Mechatronics and Energy Centre, Florida State University, Tallahassee, FL 32310-6046 USA; 4 Department of Advanced Materials and Chemical Engineering, Hanyang University (ERICA), Ansan, 15588 South Korea; 5 School of Nanoscience and Biotechnology, Shivaji University, Kolhapur, 416004 MS India; 6 Department of Pure and Applied Chemistry, University of Strathclyde, Technology and Innovation Centre, 99 George Street, Glasgow, G1 1RD UK

**Keywords:** Diseases, Health care, Chemistry, Materials science, Nanoscience and technology

## Abstract

Herein, the efficient serotonin (5-HT) sensing studies have been conducted using the (ZnO NRs)_1−x_(CNs)_x_ nanocomposites (NCs) having appropriate structural and electrochemical properties. Initially, the different compositions of ZnO nanorods (NRs), with varying content of carbon nanostructures (CNs=MWCNTs and RGO), are prepared using simple in-situ wet chemical method and thereafter these NCs have been characterized for physico-chemical properties in correlation to the 5-HT sensing activity. XRD Rietveld refinement studies reveal the hexagonal Wurtzite ZnO NRs oriented in (101) direction with space group ‘P6_3_mc’ and both orientation as well as phase of ZnO NRs are also retained in the NCs due to the small content of CNs. The interconnectivity between the ZnO NRs with CNs through different functional moieties is also studied using FTIR analysis; while phases of the constituents are confirmed through Raman analysis. FESEM images of the bare/NCs show hexagonal shaped rods with higher aspect ratio (4.87) to that of others. BET analysis and EIS measurements reveal the higher surface area (97.895 m^2^/g), lower charge transfer resistance (16.2 kΩ) for the ZCNT 0.1 NCs to that of other NCs or bare material. Thereafter, the prepared NCs are deposited on the screen printed carbon electrode (SPCE) using chitosan as cross-linked agent for 5-HT sensing studies; conducted through cyclic voltammetry (CV) and square wave voltammetry (SWV) measurements. Among the various composites, ZCNT0.1 NCs based electrodes exhibit higher sensing activity towards 5-HT in accordance to its higher surface area, lower particle size and lower charge transfer resistance. SWV measurements provide a wide linear response range (7.5–300 μM); lower limit of detection (0.66 μM), excellent limit of quantification (2.19 μM) and good reproducibility to ZCNT 0.1 NCs as compared to others for 5-HT sensing studies.

## Introduction

Nanocrystalline zinc oxide (ZnO) is ubiquitous candidate used in the various applications such as energy harvesting, sensing, supercapacitors, catalysis, electronics, biomedical, etc. This is due to its reasonable cost, ease to synthesize, direct optical band gap, a large exciton binding energy (60 meV) at room temperature, bio-compatible in nature, higher chemical stability, etc^1,2^. In comparison to its other morphologies, the ZnO nanorods (NRs) have remarkable opto-electrical properties such as ultrafast photoelectric gain, wide coverage light confinement, no grain boundaries with long-distance order, better electrochemical properties, etc. and hence these are further useful in advanced technological aspects^3,4^. To overcome the constraints as well as to tune the properties of bare ZnO NRs for uplifting the performance further, the composites formations is one of the promising routes and hence investigators are continuously using the different composite materials such as BiFeO_3_^5^, carbon nanostructures(CNs)^6^, poly(vinylidene fluoride)^7^, metal oxides^8^, metals^9^ etc. Among them, the nanocomposites (NCs) of ZnO NRs with CNs [(multiwalled carbon nanotubes (MWCNTs) or reduced graphene oxide (RGO)] are of prime importance because the synergetic interactions between the materials tune the properties of the composites to be useful for the desired applications. However, the properties of ZnO NRs based composites with CNs (MWCNTs or RGO) are greatly influenced by the synthetic protocol used. Park et al.^10^ deposited the ZnO NRs on the different 1D nanostructures by thermal chemical vapor deposition and hence the composites exhibited intense luminescence properties than that of bare materials. But, costlier instrumental facilities as well as the high temperature requirement are major problems associated with the reported method. Ye et al.^11^ showed the enhanced photoactivity and photostability for the ZnO NRs-reduced graphene oxide (rGO) nanocomposites (i.e., ZnO–rGO NCs) fabricated by electrostatic self-assembly route followed by the hydrothermal treatment. Similarly, Li et al.^12^ reported hydrothermal deposition of ZnO NRs on a CNTs film based electrodes for piezoelectric generators. Liu et al.^13^ prepared ZnO/graphene composites using the combination of a simple hydrothermal reaction and spray drying methods for high performance Li-ion batteries. However, hydrothermal protocol is not scalable conveniently for large production in nano-scale dimensions. In another research article, Liu et al.^14^ also prepared the composites of ZnO NRs with GO through colloidal coagulation effect for enhanced photocatalytic activity; however composition control as well as uniform morphology are lacking in the present route. In addition, the electrostatic connectivity between ZnO nanostructures with CNTs is one of the common shortcomings for ZnO NRs based composites and therefore these experimental constraints are to be overcome with the present simple, ease wet chemical ‘sol–gel’ approach for forming the composites of ZnO NRs with CNs (MWCNTs or RGO).

In connection to sensing studies, various approaches are available to measure serotonin (5-HT) levels and these include HPLC, capillary electrophoresis, optical methods, amalgamated tools like LC–MS, HPLC, Fluorescence, etc. However, these methods are very expensive, time consuming, hence not suitable for rapid 5-HT detection. Therefore, there is need to develop a rapid, inexpensive detection method for detection of 5-HT. Electrochemical methods have been highlighted as a viable method to address these points and develop a rapid, portable detection of a range of analytes^15–17^ including 5-HT determination primarily due to their simple design, cost effectiveness, dynamic response, good selectivity, sensitivity^18^, etc. The combinations of these approaches with different NCs for electrochemical 5-HT sensing studies have been explored. A composite film containing poly(bromocresol green), magnetic nanoparticles (Fe_3_O_4_) and MWCNTs fabricated for the electrochemical sensitive determination of 5-HT, but the complicated synthesis route is the main hurdle in the further development^19^. The ternary nanocomposite (silver/polypyrrole/copper oxide (Ag/PPy/Cu_2_O) prepared by using sonochemical and oxidative polymerization for 5-HT detection showed that chemical stability of Cu_2_O is one of the major issue and hence the present composite would not be feasible for industrial scale applications^20^. Simultaneous sensing of 5-HT and norepinephrine(NE) determined by using ionic liquid–carbon nanotubes (IL–DC–CNT) nanocomposite; however the higher limit of detection (LOD) response of this present investigation is the constraint for sensing lower concentrations of an analytes^21^. The amperometric determination of 5-HT reported using the NCs of cobalt oxide nanocubes incorporated with reduced graphene oxide (rGO–Co_3_O_4_), however the diminutive linear range and high LOD are the major laggings in the 5-HT sensing studies^22^. Along with these constraints, the other shortcomings during the NCs-based 5-HT sensing studies include thin film formation of hybrid systems, interconnectivity between the different components, interferences from other biomolecules, and robustness of the materials, etc.; which hamper the potential use of NCs for the 5-HT electrochemical sensing studies. Therefore, to overcome the constraints of NCs-based electrochemical 5-HT sensing studies, the NCs of ZnO NRs with CNs are designed using wet chemical method for rapid and selective sensing studies. To the best of our knowledge, no one reported the use of (ZnO NRs)_1−x_(CNs)_x_ NCs for 5-HT sensing studies and hence the electrochemical properties of the composites comprising ZnO NRs with CNs (MWCNTs or RGO) in relation to 5-HT sensing studies.

With these motivations as well as to overcome the constraints of NCs related to sensing studies, the attempts have been made for designing of ZnO NRs based composites with CNs using simple, ease wet chemical method and further these composites have been utilized for electrochemical 5-HT sensing studies.

## Experimental

### Synthesis of bare ZnO NRs

ZnO NRs were prepared by using simple sol–gel method. The desired amount of zinc acetate(Zn (CH_3_COO)_2_·2H_2_O and polyethylene glycol (PEG)was dissolved in double distilled water (DDW) separately and mixed with each other with constant stirring. The pH of the resulting solution was maintained up to 10.00 ± 0.02 using aqueous ammonia solution for the formation of gel. Afterthat, the gel-solution was stirred in room temperature for 2 h and the obtained precipitate was filtered, washed thrice with DDW, twice in ethanol and dried in electric oven at 80 °C up to dryness. Finally, the dried crude product was sintered at 400 °C for 2 h for getting the white crystalline powder of ZnO NRs.

### Synthesis of (ZnO NRs)_1−x_(CNs)_x_ nanocomposites

The (ZnO NRs)_1−x_(CNs)_x_ NCs with varying content of CNs [(MWCNTs from 0.1, 0.3 up to 0.5 wt%) or [(RGO from 0.04, 0.08 upto 0.1 wt%)] were prepared by using in-situ wet-chemical protocol. Initially the appropriate amount of the CNs (MWCNTs and RGO) was dispersed in 10 mL distilled water separately through ultrasonic treatment for 10 min. and directly added into the running synthetic route of ZnO NRs after the gel formation. After the addition of CNs into the gel, the white gel becomes slightly blackish indicates the incorporation of CNs with ZnO NRs. Finally, the blackish precipitate was filtered, washed then dried in electric oven at 80 °C and finally sintered at 400 °C for 2 h. The respective (ZnO NRs)_1−x_(CNs)_x_ NCs with varying the content of CNs were labeled as ZCNT0.1, ZCNT0.3, ZCNT0.5 with respect to the MWCNTs content of 0.1, 0.3, 0.5 wt%, respectively; as ZRGO0.04, ZRGO 0.08, ZRGO0.1 with respect to the RGO contents of 0.04, 0.08, 0.1 wt%, respectively.

### Modification of screen printed carbon electrodes using (ZnO NRs)_1−x_(CNs)_x_ nanocomposites

The dispersion of ZnO NRs or its NCs with CNs (MWCNTs and RGO) were prepared using the mixture of chitosan as well as acetic acid and then it was dropped on a screen printed carbon electrode (SPCE) from Dropsens (DRP-110). Thereafter, it was dried overnight at room temperature for further electrochemical analysis. The modified NCs were prepared using chitosan. 0.1 g chitosan was dissolved in 1% acetic acid. To this 3 mg of ZnO NCs was dispersed in 5 mL. Chitosan can develop a positive charge within an acidic environment^23^ and thus can be useful for dispersing and stabilizing the NCs described in this study^24,25^.

### Characterizations

X-ray diffraction analysis of bare or NCs was measured using X-ray diffractometer, with Cu Kα (1.54 Å) as the incident radiation in the 2θ range from 10° to 80°. Field emission scanning electron microscopy (FESEM) images and energy dispersive X-ray spectroscopy (EDS) mapping of the samples were obtained using a TESCAN (MIRA3). FTIR spectra of the samples were measured on Infra-Red spectrometer (Bruker Alpha FT9) in the range between 4000 and 400 cm^−1^. X-ray photoelectron spectra (XPS) of the samples were collected from X-ray photoelectron spectrometer (VG Multilab 2000-Thermo Scientific USA, K-Alpha) with a multi-channel detector which can endure high photonic energies from 0.1 to 3 keV. High resolution transmission electron microscopy (HRTEM) measurement of the representative sample was deployed from JEOL-JEM-2100F operated at 200 kV. The specific surface area of the samples was measured by the Barrett–Joyner–Halenda (BJH) method using a BET surface analyzer (Quantachrome, Autosorb-iQ, USA). Raman (FT-Raman) spectra of the samples were recorded in the spectral range of 1–5000 cm^−1^ using FT-Raman spectrophotometer (Bruker MultiRAM, Germany). Thermal Gravimetric Analysis (TGA) of the as-prepared sample was recorded in the temperature range from room-temperature–1000 °C under N_2_ flow at a heating rate of 10 °C/min. using a Perkin-Elmer thermal analyzer.

## Results and discussion

### Thermal analysis

**Figure 1:**
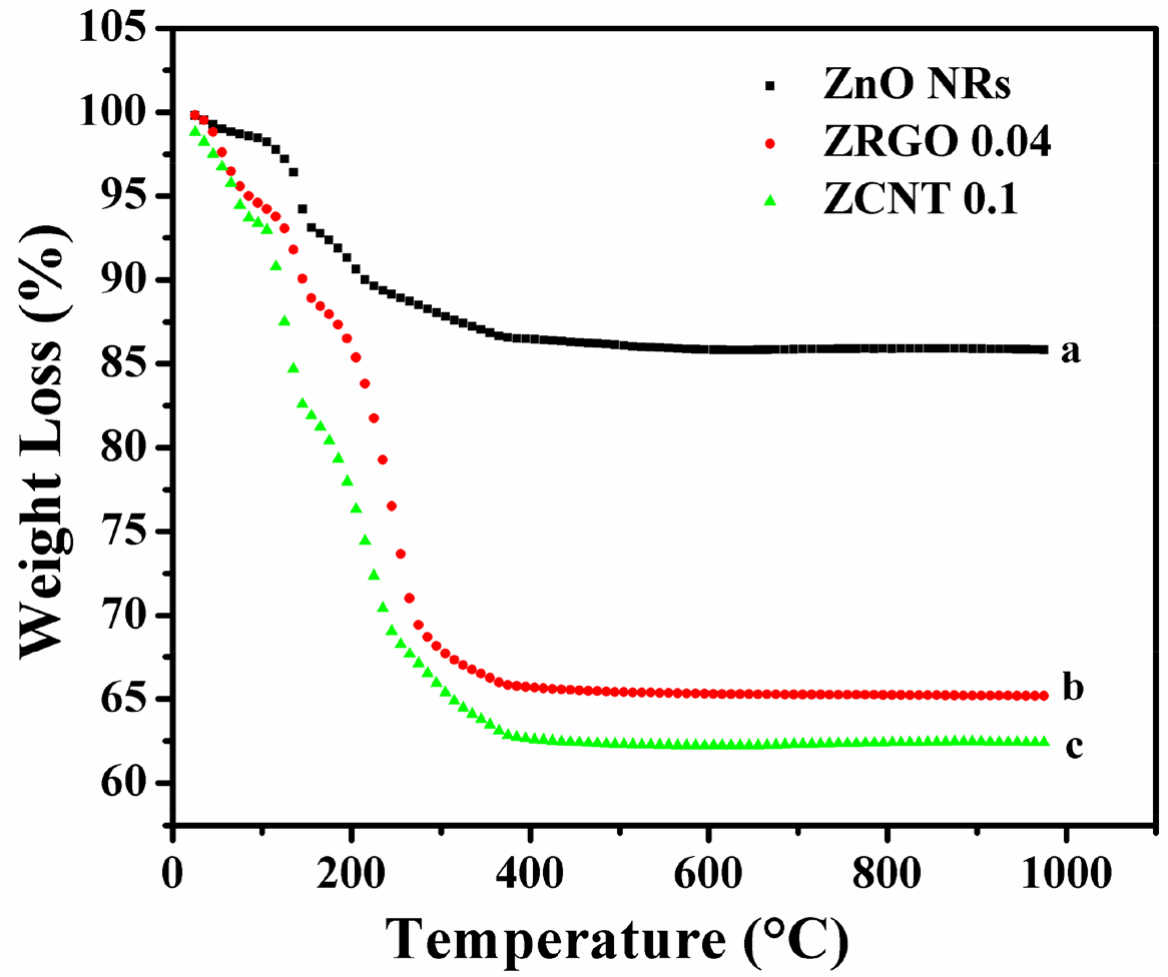
TGA thermograms of (a) ‘as prepared’ bare zinc hydroxide precursor, (b) ‘as-prepared’ bare zinc hydroxide precursor with RGO (for forming ZRGO 0.04 NCs), (c) ‘as-prepared’ zinc hydroxide precursor with MWCNTs (for forming ZCNT 0.1 NCs).

To investigate the thermal studies of ‘as-prepared’ zinc hydroxide precursor with or without CNs (MWCNTs and RGO), the thermogravimetric analysis (TGA) has been deployed. Figure 1 shows the TGA analysis of ‘as-prepared’ bare zinc hydroxide precursor, ‘as-prepared’ zinc hydroxide precursor with MWCNTs (for forming ZCNT 0.1 NCs) as well as ‘as-prepared’ bare zinc hydroxide precursor with RGO (for forming ZRGO 0.04 NCs). All the thermograms have similar nature of weight loss; consist of four stages. The details of weight losses for the different chemical moieties lost in all these samples are shown in [Table S2 Supporting Information (SI)]. The first weight loss is observed from temperature up to 125 °C due to the loss of lattice water or water of crystallization from the hydrated precursor^26^. The second weight loss is observed from 125 to 175 °C due to the loss of co-ordinated water. The drastic weight loss for third stage is observed from 175 to 400 °C is observed due to the loss of functional groups of organic moieties present in PEG as well as the oxidative decomposition of Zn-precursor and combustion of CNs from the NCs^27,28^. In fourth stage, the negligible weight loss is observed beyond 400 °C revealing the formation of stable ZnO NRs. With these observations, it is revealed that the ‘as-prepared’ zinc hydroxide precursor is converted into the desired ZnO NRs as well as (ZnO NRs)_1−x_(CNs)_x_ NCs at 400 °C onwards and hence the ‘as-prepared’ samples are annealed at 400 °C. In addition, after making the NCs of ZnO NRs with CNs (either CNTs or RGO); the NCs samples show comparatively higher weight loss to that of bare ZnO NRs; it gives clear evidences for the additional surface functional moieties present in the NCs^29–31^ and hence these interpretations are further confirmed FTIR measurements of the samples.

These additional functional moieties present in the NCs would be useful for better interconnectivity between CNs with ZnO NRs and hence which would be reflected through the proper charge transport between the components in the NCs; which is to be confirmed through EIS measurements of the samples.

### X-ray diffraction analysis

**Figure 2:**
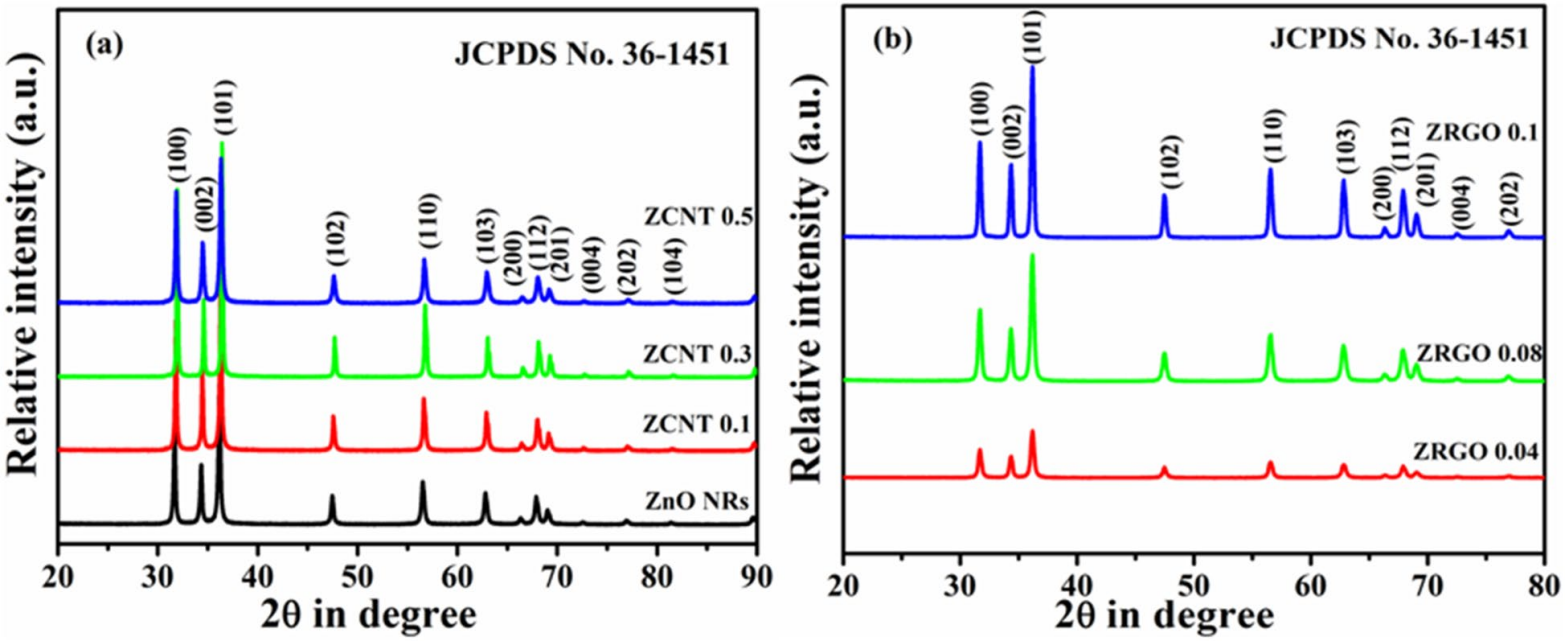
XRD diffractograms of (**a**) bare ZnO NRs and (ZnO NRs)_1−x_(CNs)_x_ NCs with varying content of MWCNTs (0.1 to 0.5 wt%) and (**b**) (ZnO NRs)_1−x_(CNs)_x_ NCs with varying content of RGO (0.04 to 1.0 wt%).

Figure 2a exhibits powder XRD patterns of bare ZnO NRs and its NCs with MWCNTs; while Fig. 2b shows XRD patterns of ZnO NCs with RGO. Powder XRD patterns of bare MWCNTs and RGO are additionally supplied in the Figure S1 (a and b SI). XRD pattern of bare ZnO NRs, shows the characteristic peaks of hexagonal wurtzite ZnO phase at 31.69°, 34.36°, 36.16°, 47.47°, 56.53°, 62.8°, 66.37°, 67.89°, 69.03°, 72.55°, 76.89°, 81.40° corresponding to the (100), (002), (101), (102), (110), (103), (200), (112), (201), (004), (202), (104) reflections; respectively and the corresponding a, c lattice constant values are 5.20 and 3.24 Å, respectively (*c*_o_/*a*_o_ = 1.60) (JCPDS 36-1451; space group P6_3_mc)^32,33^. XRD characteristics reflections of the bare ZnO material is clearly matched with that of its nanorod morphology; which is in good agreement with the earlier reported papers^10,34–39^ and hence confirming the nanorod morphology of bare ZnO materials [ICDD card No: 01-076-0704)]^40^; which further supported through FE-SEM images. Using Scherrer formula, the calculated crystallite size of the bare ZnO is around 19 nm (Table S3, SI); which also reveals the crystalline nature of the sample.

In case of (ZnO NRs)_1−x_(MWCNTs)_x_ NCs, the characteristic XRD reflections of the ZnO are retained at their respective 2θ values; without the characteristic peaks corresponding to MWCNTs due to the less content of MWCNTs in the NCs^11,41–45^. However, the intensity of characteristic XRD reflections is changed with respect to MWCNTs content in the NCs. The change in intensity of XRD peaks is ascribed with the various possible reasons such as change in atomic coordinates, crystallite size effect, cell volume, etc^46^. Along with other structural parameters, the changes in the Zn and O coordinates in the representative materials are confirmed through the Rietveld refinement data (shown in Table 1). This data reveals that the atomic positions of Zn and O in the NCs are somewhat different to that of bare ZnO materials. In connection to crystallite size, the increase in degree of crystallinity of the NCs also reflected through the change in the X-ray intensity of the reflections; which is shown in table [Table S3 (SI)]^46^. Similar results are also observed with unit cell volume that displays the scattering power and hence directly correlating to the intensity of incident X-rays per volume; which is shown in Table 1^46^. In XRD diffractograms of (ZnO NRs)_1−x_(RGO)_x_ NCs, there is no change in the position of peaks of the characteristics ZnO reflections; however the intensity of these reflections is somewhat decreased with increase in the RGO content in the NCs^47^. The decrease in intensity of the characteristics reflections is supported through the presence of low X-ray scattering power of RGO in the NCs^11,42^.

**Table 1:** Rietveld Refinement structural parameters of ZnO NRs and ZCNT 0.1 NCs.

Parameters	ZnO NRs	ZCNT 0.1 NCs
Space group	Hexa (P6_3_mc)	Hexa (P6_3_mc)
a (Å)	3.24735(3)	3.24800(3)
c (Å)	5.20223(8)	5.20324(8)
Vol (Å^3^)	55.20223(1)	54.89160(1)
Zn (x, y, z)	0.33333	0.33333
	0.66667	0.66667
	0.00000	0.00000
B (Å^2^)	0.855(23)	1.097( 27)
O (x, y, z)	0.33333	0.33333
	0.66667	0.66667
	0.38082(48)	0.38272(55)
B (Å^2^)	0.922(89)	0.766(102)
R-factors and χ^2^	R_P_: 6.40	R_P_: 7.51
	R_wp_: 9.29	R_wp_: 10.5
	R_exp_: 6.81	R_exp_: 6.53
	χ^2^: 1.86	χ^2^: 2.57
R_B_	2.85	3.75
R_F_	2.33	3.59

**Figure 3:**
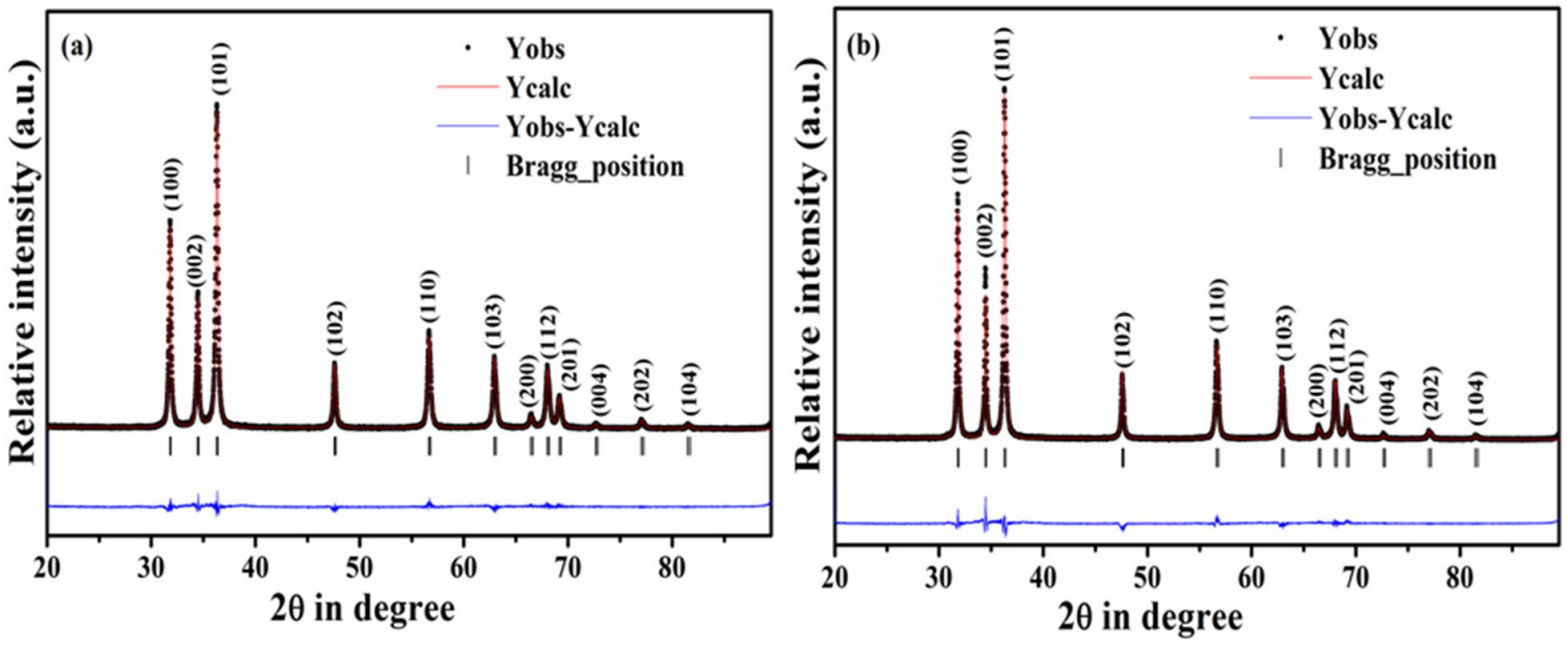
XRD Rietveld refined patterns of (**a**) bare ZnO NRs with calculated pattern (red), observed pattern (black), the difference between calculated and observed pattern (blue); (**b**) ZCNT 0.1 NCs with calculated pattern (red), observed pattern (black), the difference between calculated and observed pattern (blue).

The relative intensity ratio (RIR) analysis of the characteristics peaks of bare ZnO NRs and NCs with varying content of MWCNTs and RGO are presented. The significant changes in the intensity and shift in 2θ values are studied through this analysis (See 3.2 SI). The detailed quantitative studies about the structural parameters of the representative ZnO NRs and ZCNT 0.1 NCs are further confirmed through the Rietveld refinement method using the Fullprof 2000 software package and hence Rietveld refined XRD patterns of the representative ZnO NRs and ZCNT 0.1 NCs are shown in Fig. 3a,b. In the refinement, the oxygen positions (x, y, z) have been considered as free parameters, and fractional atomic positions have been taken as fixed. During refinements, potential profile broadening due to strain and defects are not considered. All Rietveld refinement factors such as, goodness of fit (χ^2^), and various R-factors such as R_p_ (profile factor), R_wp_ (weighted profile factor), R_exp_ (expected factor), R_B_ (Bragg factor), R_F_ (crystallographic factor) along with the lattice parameters and unit cell volume with errors (in brackets) are summarized in the Table 1.

The refined as well as observed XRD patterns of ZnO NRs and ZCNT 0.1 NCs are fitted well due to negligible difference between Y_*i*_^*obs*^ with Y_*i*_^*calc*^ and hence it has also been reflected through either a visual judgment as straight blue line parallel to the horizontal axis or good agreement of the refined structural parameters (seen in Table 1) with the calculated structural parameters of the respective materials [seen in Table S3 (*SI*)]. χ^2^ value is determined with the help expected and weighted profile R-factors using the equation: χ^2^ = (R_wp_/R_exp_)^2^ and this value is around 2.0 for both the samples revealing high quality of the fit; which is in close agreement with the reported paper^48,49^. R_B_ and R_F_ indices of the ZCNT 0.1 NCs are somewhat higher to that of ZnO NRs revealing the peaks of the ZCNT 0.1 NCs have very long tails or significant unmodeled asymmetry, because parts of the peak are not included in the intensity estimate. In addition, R_B_ factor of the both samples is less than 5.00 indicating the proper fitting of the structural parameters of refined database to that of observed database of the bare materials^49,50^.

### FTIR analysis

**Figure 4:**
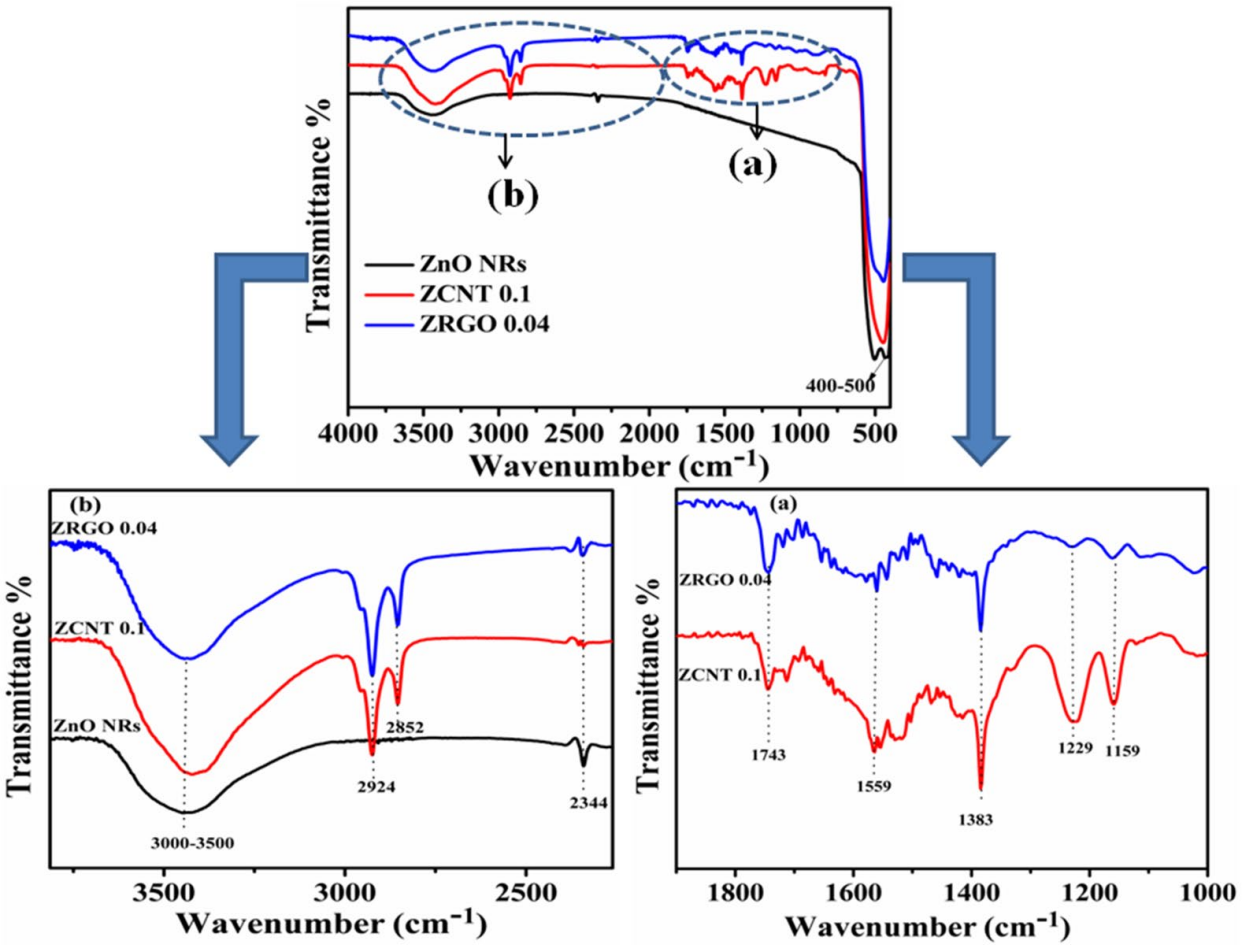
FTIR spectra of bare ZnO NRs, representative ZCNT 0.1 and ZRGO 0.04 NCs (**a**, **b**) with their enlarged views.

Figure 4 displays the FTIR spectra of the bare ZnO NRs, representative ZCNT0.1 NCs and ZRGO0.04 NCs. FTIR spectrum of bare ZnO NRs consists of three characteristics transmission vibrational bands viz 400–700 cm^−1^ due to Zn–O stretching mode, 2343 cm^−1^ due to atmospheric CO_2_ and broad frequency region at 3000–3500 cm^−1^ due to–OH functional moieties^29,34,51^. In FTIR patterns of the NCs, along with the characteristics bands, the additional bands are observed at ~ 1159, ~ 1229, ~ 1383, ~ 1559, ~ 1743 (Fig. 4a), ~ 2852, ~ 2924 cm^−1^ (Fig. 4b) corresponding to the C–O (ester) stretching vibrations^52^, C–OH stretching vibrations from carboxylic group (–COOH)^53^, C=C stretching from unoxidized graphitic area, carbonyl group (C=O)stretching vibrations^52,53^, asymmetric as well as symmetric C–H stretching frequency vibrations originated from CNs (MWCNTs and RGO) respectively^53^. As compared to the bare ZnO NRs as well as CNs, the characteristics IR bands are slightly shifted revealing the chemical interconnectivity between the surface hydroxyl (–OH) functional group of ZnO NRs with acidic (–COOH) group of MWCNTs or RGO. In addition, it is seen that the broad –OH stretching band for ZCNT 0.1 NCs as compared to bare or other NCs due to the more number of –OH groups at the surfaces; which would have distinct effects in the improvement of the electrochemical activity as well as electronic charge transport properties of the desired NCs^54^.

### Raman analysis

**Figure 5:**
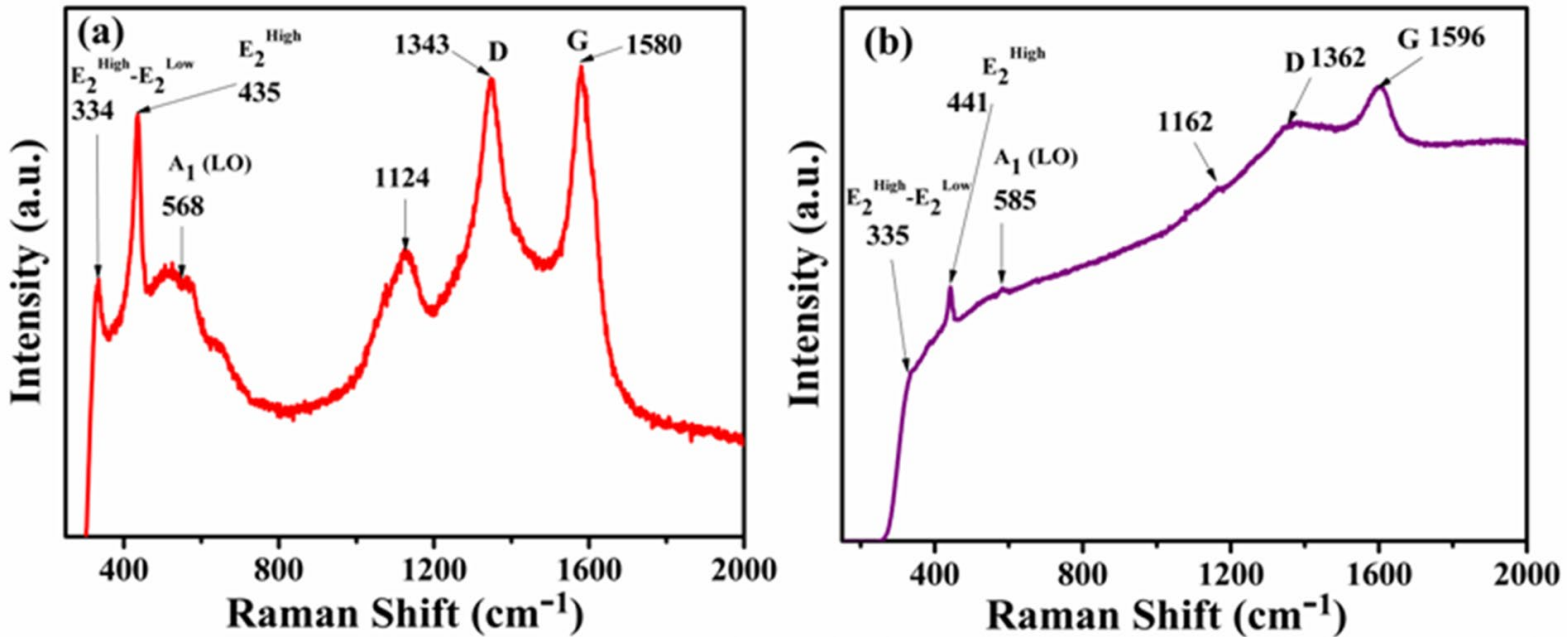
Raman spectra of the representative (**a**) ZCNT 0.1 and (**b**) ZRGO 0.04 NCs.

The Raman spectra for bare ZnO NRs, MWCNTs as well as RGO are shown in Figure [Figure S4 (a) SI] and these spectra are in good agreement with the results reported elsewhere^26,55–58^. Figure 5a,b consists of Raman spectra for the representative ZCNT 0.1 and ZRGO 0.04 NCs. A spectrum of ZCNT 0.1 NCs shows the characteristics Raman bands of ZnO NRs (strong peak at 435, 1124, 568, 334 cm^−1^) as well as MWCNTs (1343, 1580 cm^−1^); which reveal the presence of the desired components in the NCs. The strong peak at 435 cm^−1^ corresponds to E_2_^High^ (high frequency optical mode) confirming the presence of hexagonal wurtzite phase in accordance to the XRD interpretation. The remaining peaks at 568, 334, 1124 cm^−1^ analogous to the A_1_ longitudinal optical (LO) mode which is related to the defects either due to the oxygen vacancies or Zn(II) ions interstitials from the lattice ZnO, E_2_^High^ − E_2_^Low^ second order modes of vibrations and multiple-phonon scattering processes, respectively^34,55^.

Figure 5b consists of Raman spectrum of ZRGO 0.04 NCs and the observed results for ZRGO 0.04 NCs are similar to that of ZCNT 0.1 NCs. However, slight change in the peak positions of the characterstic Raman bands is observed in the NCs to that of bare materials; which also reveal the surface functional group interaction between ZnO NRs and CNs. The positions of the two characteristics bands corresponds to the D (indicative disorder graphitic structure) and G (ordered graphitic structure) bands of CNs with their intensities ratio for the samples are also included in supporting information (Table S4, SI)^59,60^.

### FESEM analysis

**Figure 6:**
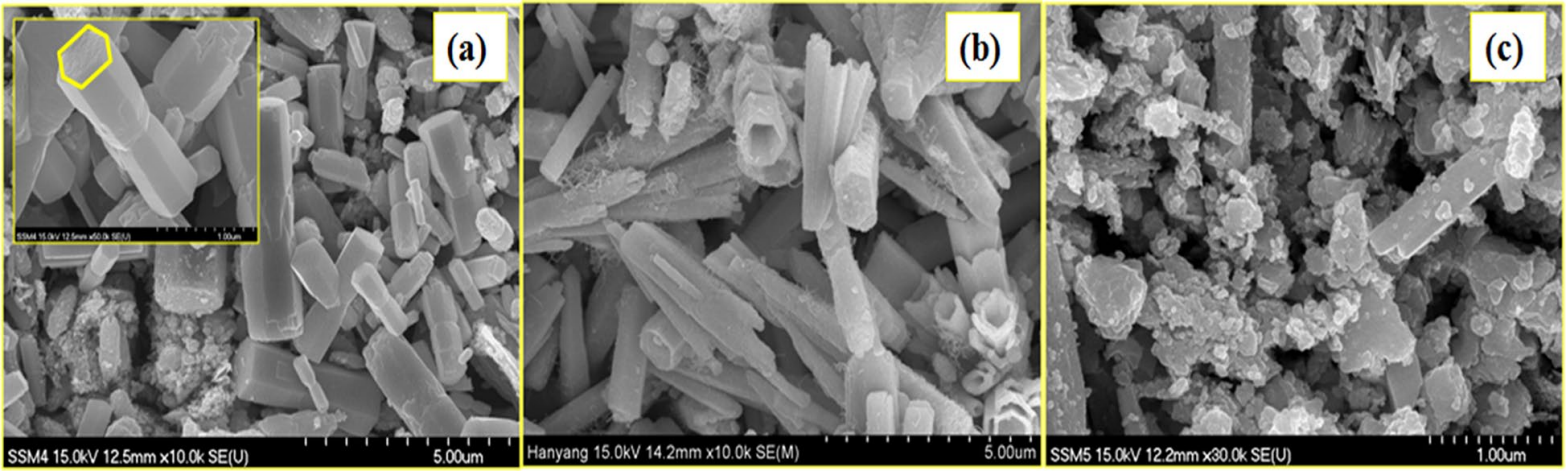
FESEM images of the (**a**) bare ZnO NRs, (**b**) ZCNT 0.1, (**c**) ZRGO 0.04 NCs.

To investigate the topographical features of the ZnO NRs and its representative (ZnO NRs)_1−x_(CNs)_x_ NCs, the field emission scanning electron microscopy (FESEM)has been employed. FESEM image of the bare ZnO NRs (Fig. 6a) shows the well distributed clear hexagonal NRs having average outer hexagonal diameter of 682 nm and an average length of 2.36 μm with an aspect ratio 3.45. In FESEM image of ZCNT 0.1 NCs (Fig. 6b), good interconnectivity between ZnO and MWCNTs is observed. In addition, ZnO NRs dimensions are orderly as compared to others; which are reflected through the crystallographic parameters of an average outer hexagonal diameter of 521 nm and an average length of 2.54 μm with an aspect ratio 4.87. In ZRGO 0.04 NCs (Fig. 6c), the connectivity between ZnO NRs with RGO is not upto the mark due to agglomerated materials. The average outer hexagonal diameter of 1414 nm, length of 0.875 μm with an aspect ratio 0.61 in ZRGO 0.04 NCs is observed. Due to a high aspect ratio, the ZCNT 0.1 NCs would be having higher surface loading or immobilization of the biomolecule resulting in better electrochemical sensing studies of 5-HT at the SPCE.

### EDS analysis

**Figure 7:**
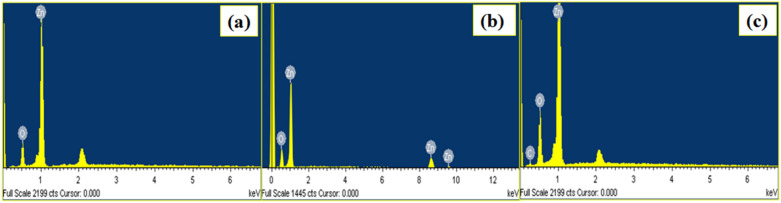
EDS spectra of the (**a**) bare ZnO NRs, (**b**) ZCNT 0.1 NCs, (**c**) ZRGO 0.04 NCs.

The quantitative elemental analysis of the bare ZnO NRs and its (ZnO NRs)_1−x_(CNs)_x_NCswas studied through the energy dispersive X-ray spectroscopy (EDS) measurements. Figure 7a–c consists of the EDS patterns for bare ZnO NRs and its representative (ZnO NRs)_1−x_(CNs)_x_ NCs. Figure 7a shows the characteristics peaks of the elemental Zn and O and mislaid of the other peaks in the all patterns indicating no other impurities present in the prepared ZnO NRs. Also, the Fig. 7b,c shows the exhibition of all the characteristics peaks corresponding to the elemental Zn, elemental O as well as elemental C revealing the presence of these elements in the NCs of wurtzite ZnO NRs with CNs (MWCNTs and RGO). The observed elemental compositions of the bare ZnO NRs and its (ZnO NRs)_1−x_(CNs)_x_ NCs are illustrated in Table S5 (Table S5, SI). From the Table S5 it is seen that, the observed elemental compositions (wt%) in the NCs are well match with the theoretical predictions.

### XPS analysis

**Figure 8:**
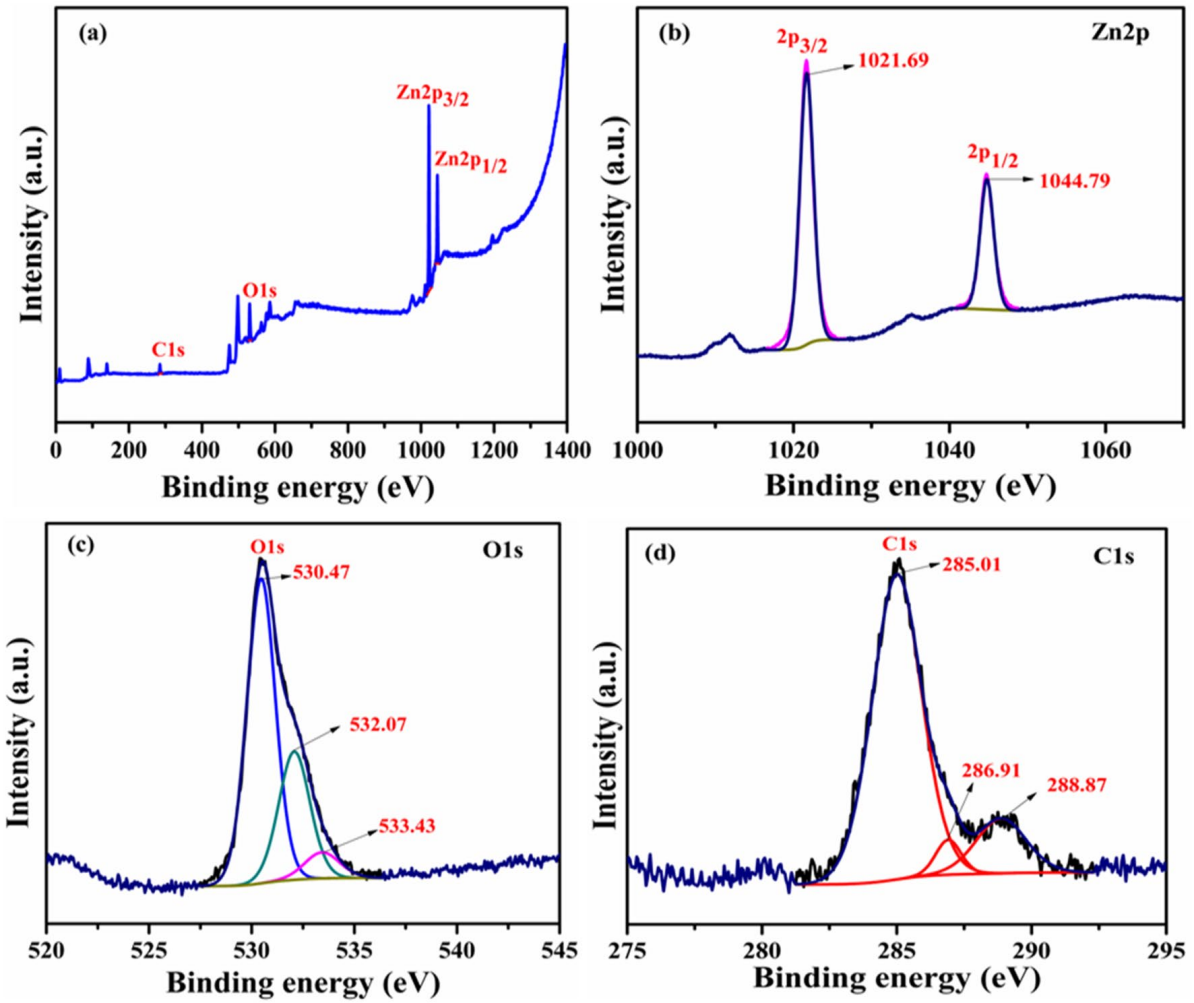
(**a**) XPS survey spectrum and high resolution core level XPS spectrums of (**b**) Zn 2*p* (**c**) O 1*s* and (**d**) C 1*s* of the representative ZCNT 0.1 NCs.

Figure 8a shows the XPS survey spectrum of the representative ZCNT 0.1 NCs, showing the existence of the elemental compositions of Zn, O and C in the ZCNT 0.1 NCs; while the absence of other peaks in the NCs indicates that no impurities are present in the NCs. The high resolution core level XPS spectrum for Zn present in the NCs is illustrated in the Fig. 8b. It shows the appearances of the two distinct characteristics peaks at 1021.69 eV and 1044.79 eV corresponding to the Zn 2*p*_3/2_ and Zn 2*p*_1/2_, respectively. However, it shows the slight shifting of the characteristics peaks to the higher binding energy (1021.57 eV and 1044.67 eV) values to that of bare ZnO NRs [Figure S7 (b), SI]. This is attributed to the good chemical interconnectivity between the ZnO NRs and MWCNTs in the NCs. However, both the samples showing the binding energy difference between the Zn 2*p*_3/2_ and Zn 2*p*_1/2_ peaks around 23.1 eV, indicates the good evidences for the presence of Zn(II) ions in the both bare ZnO NRs and ZCNT0.1 NCs^61,62^. Figure 8c shows the high resolution core level O1*s* XPS spectrum of the representative ZCNT0.1 NCs and hence it shows the strong peak at 530.47 eV assigned due to the characteristic binding energies for lattice oxygen of ZnO and again it deconvoluted into two peaks at 532.07 eV and 533.13 eV corresponding to chemisorbed water and C–OH moieties originating from elemental oxygen^63,64^. The high resolution O1*s* XPS spectrum of bare ZnO NRs [Figure S7 (c), SI] shows no significant changes after the incorporation of MWCNTs into the ZnO host lattice. Figure 8d shows the high resolution core level C1*s* XPS spectra of the ZCNT 0.1 NCs and it shows the main peak at 285.01 eV attributed due to the sp^3^ defect and other deconvoluted peaks at 286.91 eV and 288.87 eV corresponding to hydroxyl(–OH) group and carboxyl (O=C–O) functional moieties respectively from the MWCNTs incorporated with ZnO NRs^61,62,65–67^.

**Figure 9:**
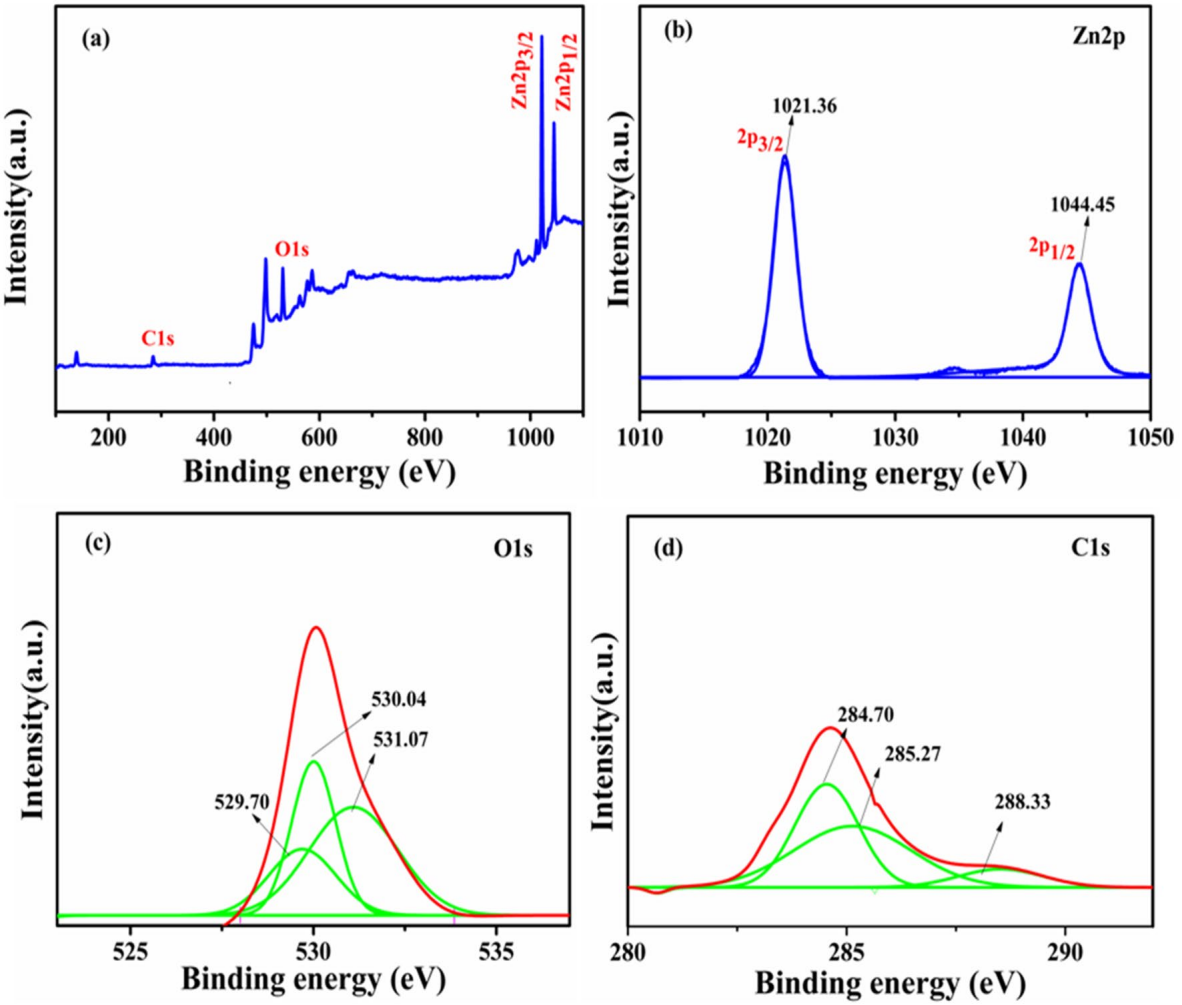
(**a**) XPS survey spectrum and high resolution core level XPS spectrums of (**b**) Zn 2*p* (**c**) O 1*s* (**d**) C 1*s* of the representative ZRGO 0.04 NCs.

Figure 9a shows the XPS survey spectrum of the ZRGO 0.04 NCs; while Fig. 9b–d show the high resolution core level XPS spectra of the Zn 2*p*, O1*s* and C1*s* of the representative ZRGO 0.04 NCs; respectively. The observed results suggest good agreement with the results obtained from the ZCNT 0.1 NCs. However, slight change in the peak positions is observed to that of ZCNT 0.1 NCs, which is due to the differences in the defects from the functional moieties of MWCNTs and RGO [Figure S5 (a to c) and Figure S6 (a to c SI)] interconnected with ZnO NRs respectively^68,69^. All evidences clearly indicate the good chemical interconnectivity due to surface functional group interaction between ZnO NRs and CNs.

### BET analysis

The specific surface area of the desired bare ZnO NRs and its NCs after the incorporation of CNs (MWCNTs and RGO) was investigated by using Brunauer–Emmett–Teller (BET) analysis through adsorption–desorption of N_2_ with its relative pressure in the range of 0.01 < P/P0 < 1. Also, the total pore volume, average pore diameter and distribution of pore size of the samples were also investigated by using the Barrett–Joyner–Halenda (BJH) method.

**Figure 10:**
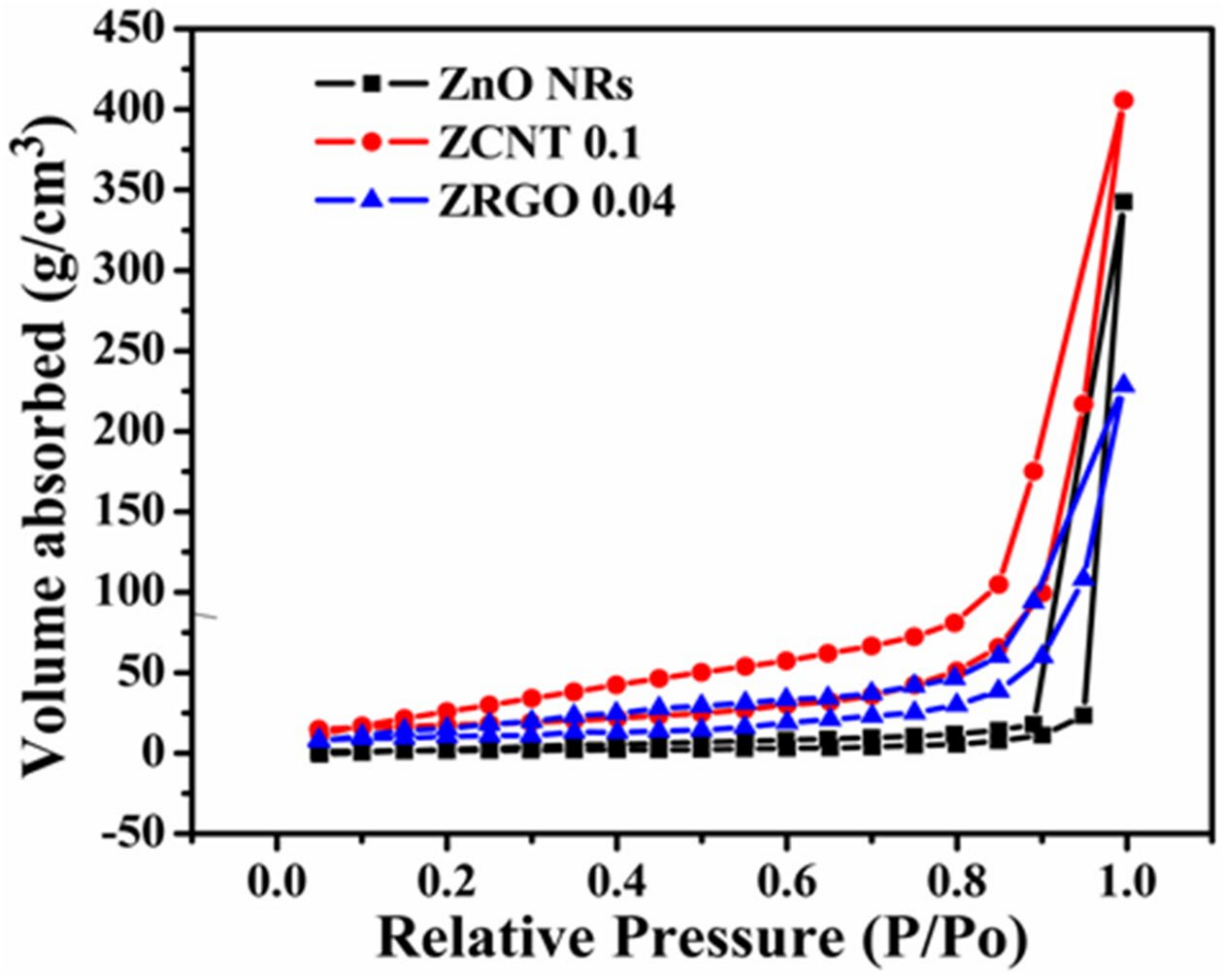
N_2_ adsorption–desorption isotherms of the bare ZnO NRs, and representative ZCNT 0.1 and ZRGO 0.04 NCs.

Figure 10 depicts the N_2_ adsorption–desorption isotherms of the bare ZnO NRs and representative ZCNT 0.1 and ZRGO 0.04 NCs. From the N_2_ adsorption–desorption isotherms, all the samples exhibits the type IV isotherms due to the formation of multilayers and type H3 hysteresis loop^70–72^. The specific surface area of the desired bare ZnO NRs, and representative ZCNT 0.1 NCs and ZRGO 0.04 NCs was calculated by using BET method and the observed values are 16.699, 97.895 and 55.078 m^2^/g, respectively (Table S6, SI). In BET measurements, the unexpected results are noted as usually decrease in surface area is observed for the NCs to that of bare materials. The increase in surface area for the ZCNT 0.1 NCs could be attributed due to the rolling structure of MWCNTs adsorbing more amount of N_2_ gas in its cavities to that of bare ZnO NRs only. On the basis of BJH measurements, pore size of the samples exhibits in the range between 2 and 50 nm indicating that the desired materials have mesoporous nature (Table S6, SI). Among these, ZCNT 0.1 NCs shows enlarged specific surface area with good porosity and hence which is supposed to enhance sensing activity towards the respective analyte.

### Electrochemical impedance spectroscopy (EIS) studies

**Figure 11:**
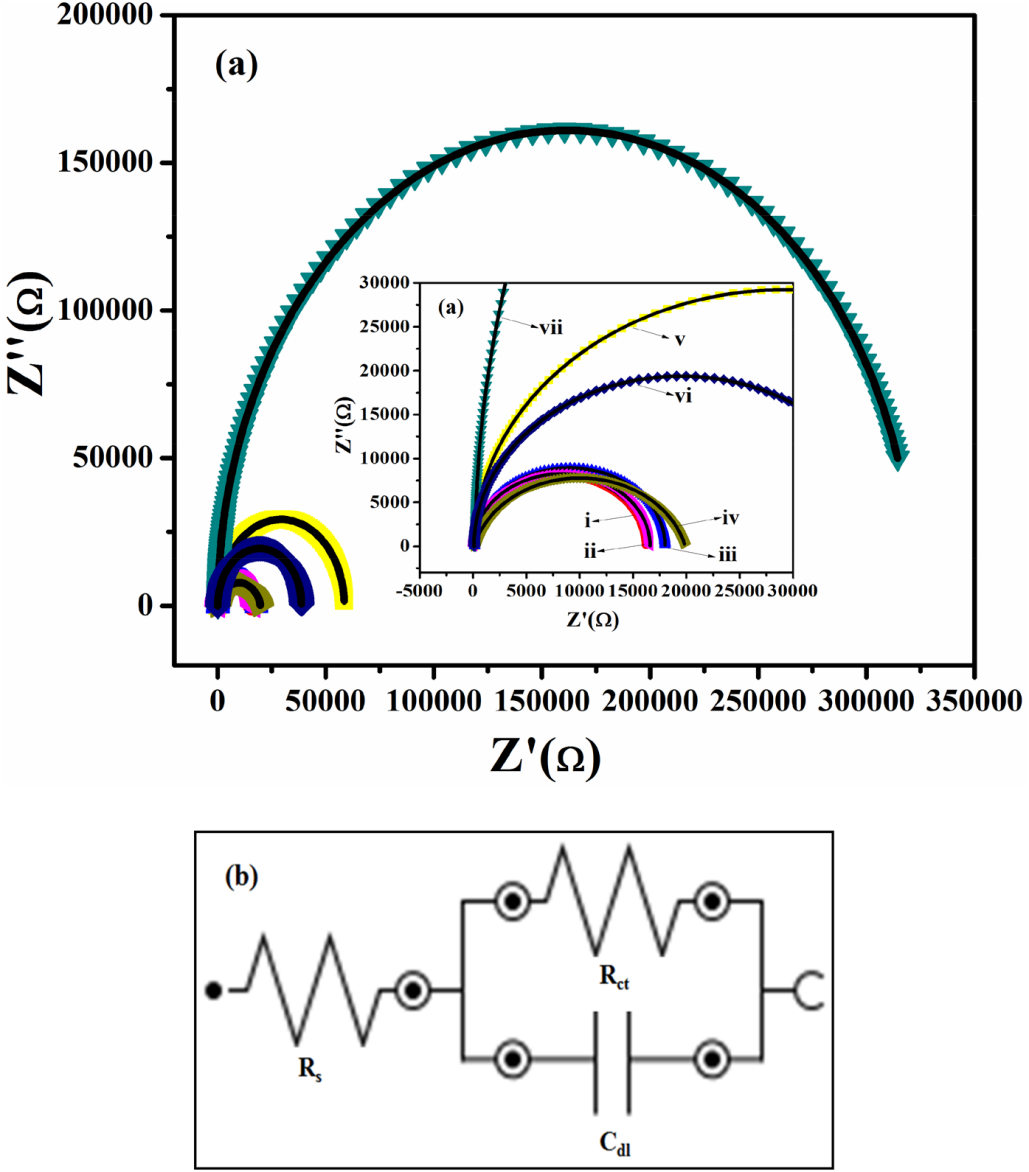
(**a**) Nyquist plots obtained for the modified electrodes (i) SPCE/ZCNT 0.1, (ii) SPCE/ZRGO 0.04, (iii) SPCE/ZCNT 0.3, (iv) SPCE/ZRGO 0.08, (v) SPCE/ZnO NRs, (vi) SPCE/ZRGO 0.1, (vii) SPCE/ZCNT 0.5 in 5 mM K_3_ [Fe (CN)_6_] and in 0.1 M PBS (pH 7.4) versus Ag wire reference electrode (symbols representing the experimental data whereas; solid line on the symbols represents the fitted data) and (**b**) model circuit used for fitting the impedance data.

The redox electrochemical impedance spectroscopy (EIS) studies were carried out to monitor the electron transfer rate between the modified electrodes and electrolyte (ferrocene redox couple). The impedance spectra (Nyquist plots) obtained for the electrodes are presented in Fig. 11a,b; which show the equivalent circuit used to fit impedance data. In Fig. 11a symbols are representing the experimental data; whereas solid line on the symbols represents the fitted data. The measured EIS data fits with the NOVA 2.1.4 software to obtain charge transfer resistance (R_ct_) of the electrodes; which is represented in the Table 2.

**Table 2:** Charge transfer resistance (R_ct_) values for the various electrodes in 5 mM K_3_[Fe(CN)_6_] and in 0.1 M PBS (pH 7.4) versus Ag electrode.

Electrodes	R_ct_ (kΩ)
SPCE/ZnO NRs	58.70
SPCE/ZCNT 0.1	16.08
SPCE/ZCNT 0.3	17.88
SPCE/ZCNT 0.5	314.54
SPCE/ZRGO 0.04	16.38
SPCE/ZRGO 0.08	19.99
SPCE/ZRGO 0.1	38.84

From table, it is seen that bare ZnO NRs having 58.70 kΩ of R_ct_ value. With the addition of either MWCNTs or RGO in the ZnO NRs, the diameter of the semi-circle with R_ct_ value for the respective electrodes is diminished to that of bare ZnO NRs, except SPCE/ZCNT 0.5 NCs. Among (ZnO NRs)_1−x_(MWCNTs)_x_ NCs, the lowest R_ct_ value (16.08 kΩ) is observed in SPCE/ZCNT 0.1 electrode, which further increases to 314.54 kΩ for SPCE/ZCNT 0.5 electrode and hence proper as well as fast exchange of charge transfer between the electrode and electrolyte observed for SPCE/ZCNT 0.1 electrode. In addition, it has also been reflecting through the higher current response (1.265 μA) to SPCE/ZCNT 0.1 NCs electrode to that of others (shown in Fig. 14).

The observed results suggest the ZCNT 0.1 NCs would have the better sensing response for the desired analyte as compared to others and hence taking into these considerations, ZCNT 0.1 NCs based electrodes are focused primly for further research endeavors through cyclic voltammetric sensing investigations of 5-HT.

### Cyclic voltammetry (CV) and square wave voltammetry (SWV) measurements

**Figure 12:**
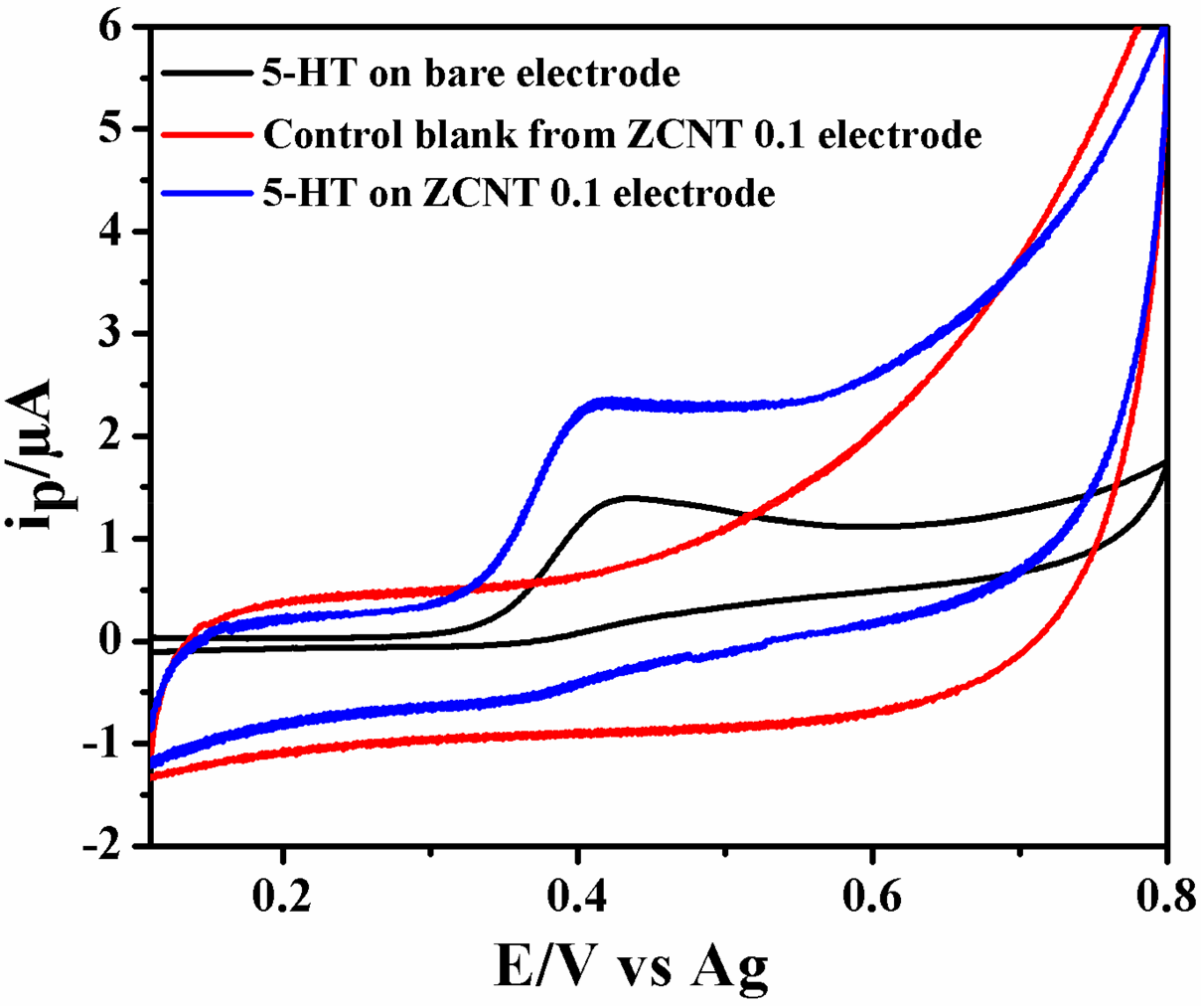
Cyclic voltammetry (CV) plot of bare SPCE (red line), 5-HT on SPCE (black line) and 5-HT on ZCNT 0.1 NCs deposited on SPCE (blue line) [0.1 M PBS (pH 7.4) scan rate: 0.02 V s^−1^).

The electrochemical detection of 5-HT using a SPCE modified with ZnO NRs and its NCs is performed using three-electrode cyclic voltammetry (CV) system^73^. Initially, the cyclic voltammogram was obtained using bare or modified SPCE/ZCNT 0.1 electrode without or with 5-HT biomolecule (40 μL, 1 mM) using PBS (0.1 M) as electrolyte and it is seen in Fig. 12. The voltammogram has been performed in the range + 0.1 to + 0.8 V versus Ag (scan rate 0.02 V s^−1^).

A well-defined oxidation peak (Fig. 12) for 5-HT is observed at + 0.437 V for SPCE electrode; while in case of SPCE/ZCNT 0.1 (modified) electrode, the oxidation peak is shifted to + 0.410 V.

Figure 12 shows the electrochemical behavior of the SPCE with an oxidation at + 0.437 V versus Ag. The oxidation peak observed for the SPCE/ZCNT 0.1 compared to the bare electrode is larger with a slight shift in the oxidation potential to 0.410 V versus Ag illustrating that the modified electrode enhances the electron transfer and peak currents of 5-HT similar to that reported for Fe_3_O_4_ nanoparticle modified electrodes^19^. The slight reduction in oxidation potential observed, maybe due in part to better diffusion of the analyte through the more porous modifying layer or possibly a minor mediated oxidation by the modifying layer^17,74–76^. This peak can also be attributed to the irreversible oxidation of the hydroxyl group of the aromatic ring of the 5-HT involving 2 electrons, to form the final ketone product^20,77,78^.

**Figure 13:**
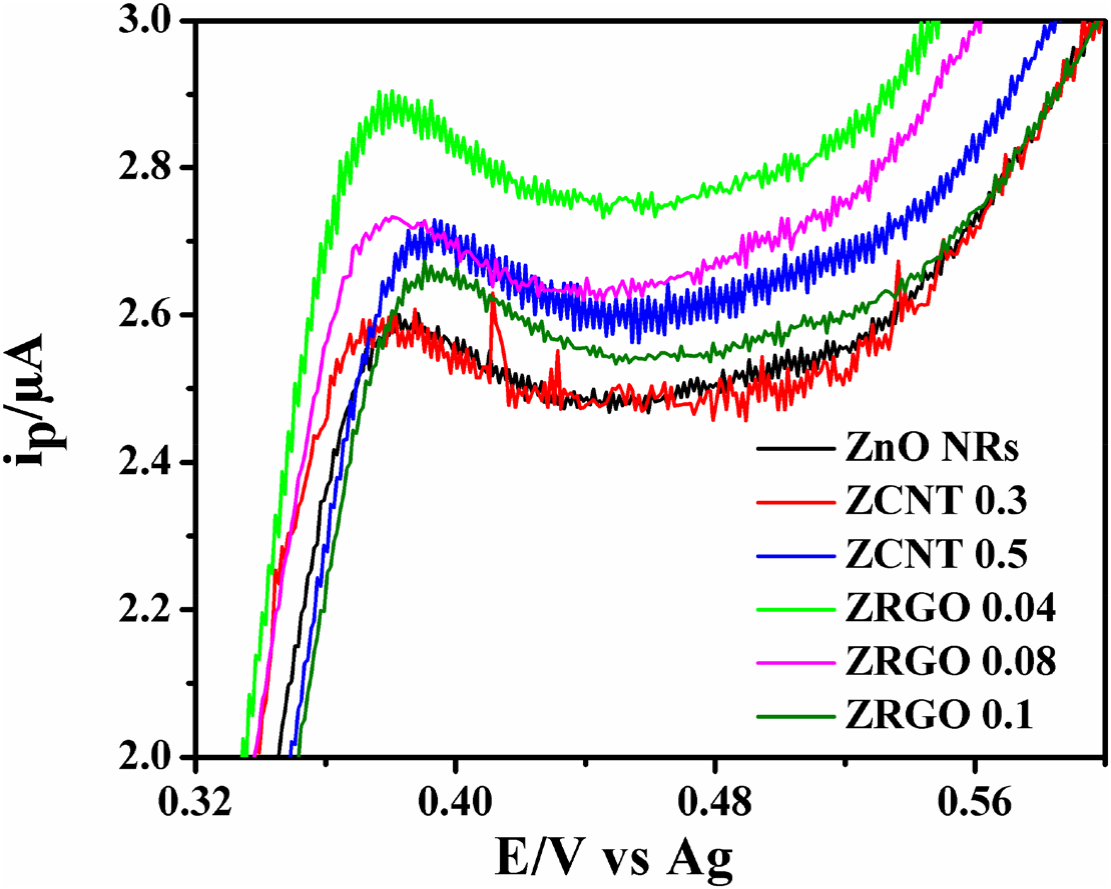
Cyclic voltammograms of SPCE/(ZnO NRs)_1−x_(CNs)_x_ NCs with their enlarged view [0.1 M PBS (pH 7.4) scan rate: 0.02 V s^−1^)].

**Figure 14:**
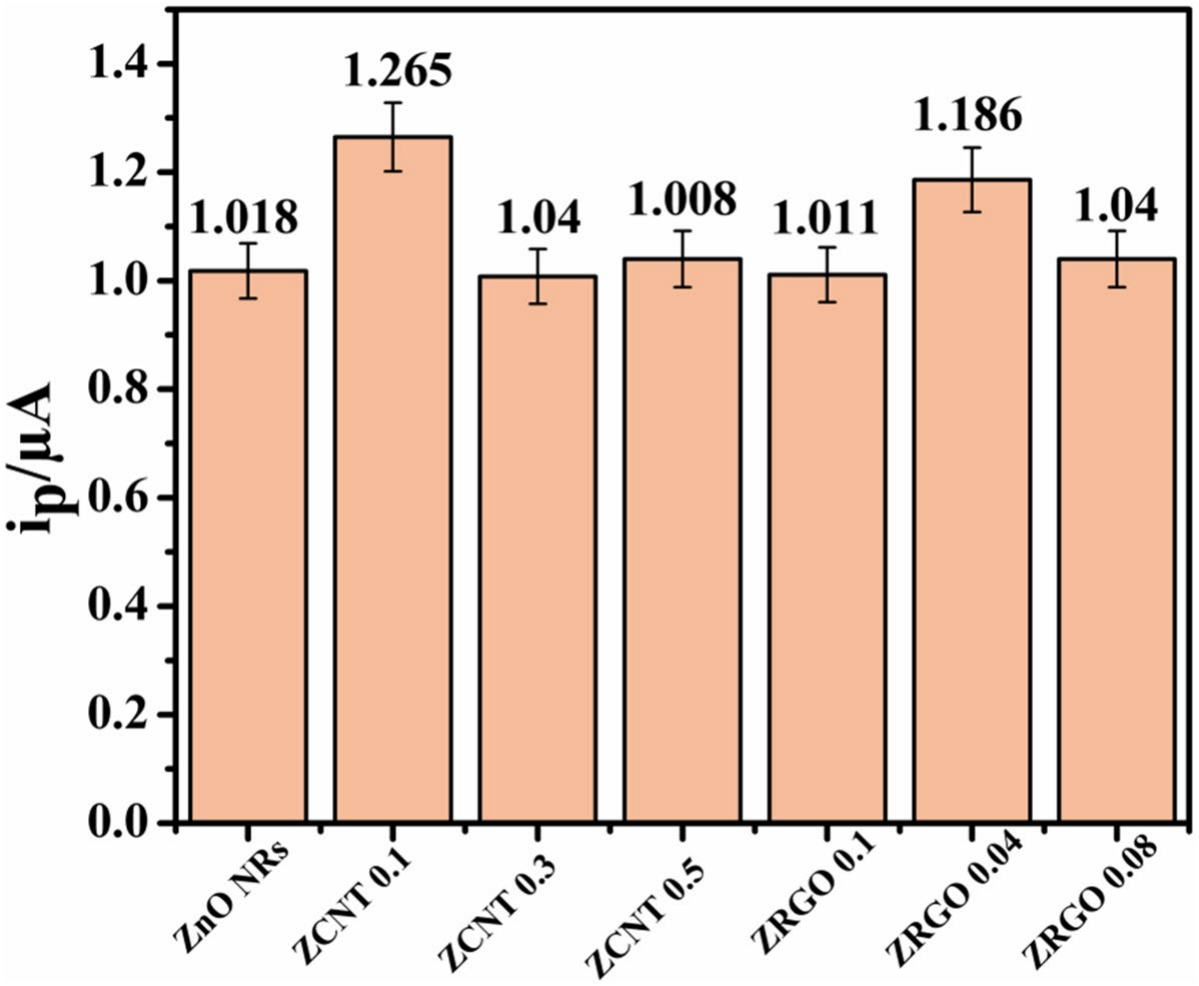
Oxidation peak signals for 1 mM 5-HT for each modified SPCE observed during the cyclic voltammetry experiment [0.1 M PBS (pH 7.4) scan rate: 0.02 V s^−1^)].

In addition, as it can be observed on the CV (Fig. 12), the modified electrodes have an enhanced current response revealing the improvement in electrical behavior of the SPCE with ZCNT 0.1 NCs based electrode for 5-HT sensing studies in comparison to the non-modified electrode. The increase in electrochemical current response for SPCE/ZCNT 0.1 electrode is attributed to improved charge transfer process as well as separable electron transfer through its lower charge transfer resistance, more electroactive area of the electrode due to smaller particle size as well as higher surface area for sensing studies. Therefore, significant sensing of 5-HT is observed for the representative ZCNT 0.1 NCs based electrodes as compared to others. In addition, from EIS data (Table 2) it is clearly seen that the R_ct_ for ZCNT 0.1 NCs is lower than that of ZGRO NCs. This indicates that the ZCNT 0.1 NCs is more conductive, supporting the observations made in Fig. 12. To know the electrochemical 5-HT sensing behavior of other NCs-based electrodes, the similar optimized cyclic voltammetry studies of SPCE/ZCNT 0.1 electrode have been conducted and the respective all NCs based electrode CVs (enlarged view) are shown in Fig. 13. With increase in content of MWCNTs in the (ZnO NRs)_1−x_(MWCNTs)_x_ NCs, there is little variations in the current response for 5-HT sensing studies and it is observed in the range between 1.018 and 1.008 μA. In case of RGO based NCs, the higher current response for 5-HT sensing studies is observed for ZRGO 0.04 NCs. The current response value of the samples is shown in Fig. 14. Hence, in all NCs based electrodes, the most significant 5-HT sensing activity is noted for SPCE/ZCNT 0.1 electrode and for further experiments SPCE/ZCNT 0.1 NCs will be used.

**Figure 15:**
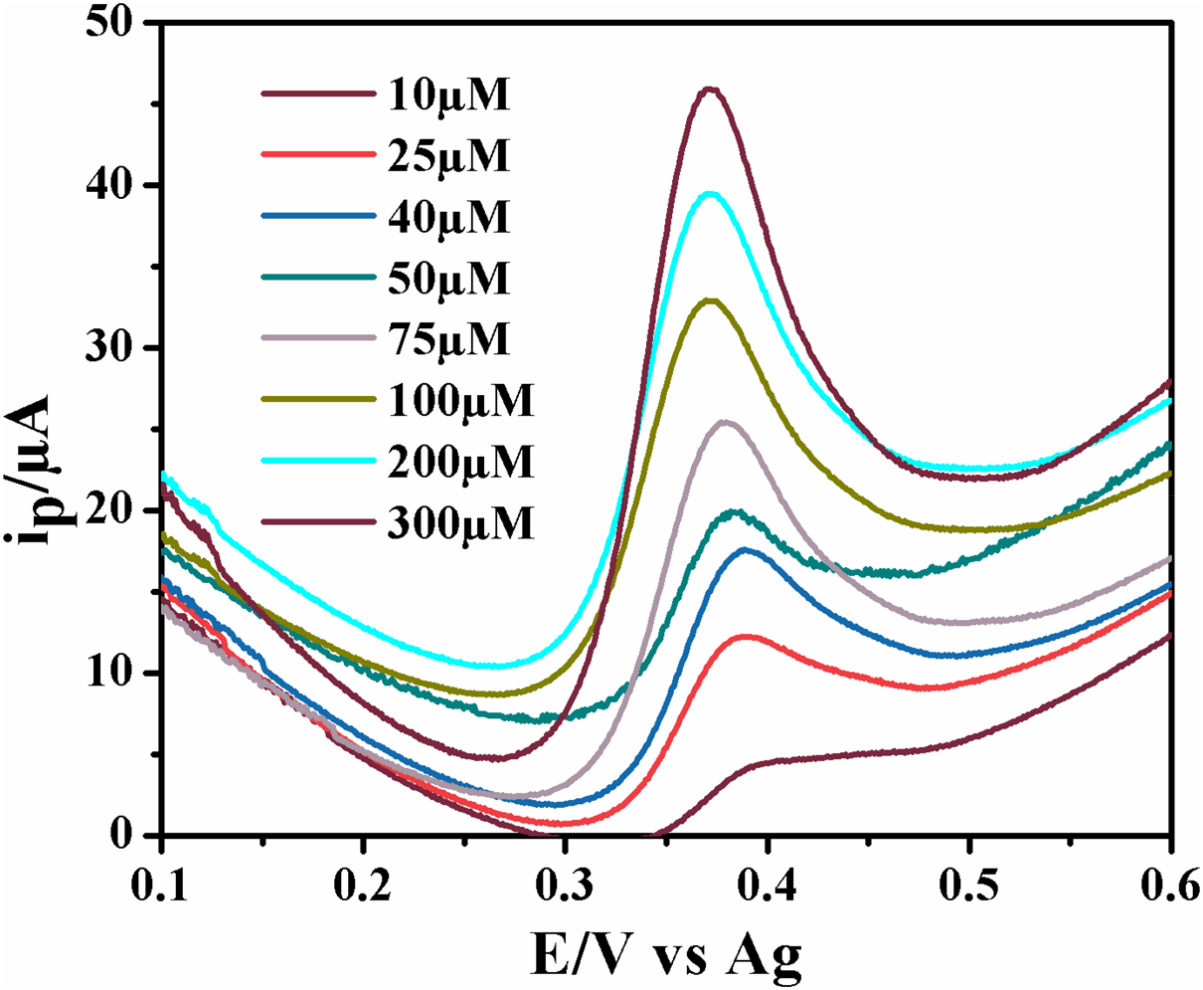
SWV response (Estep: 0.001 V; Amplitude: 0.05 V; Frequency: 25 Hz) from the ZCNT 0.1 NCs modified SPCE with the concentration of 10 to 300 μM 5-HT.

To verify the applicability of the ZCNT 0.1 NCs modified SPCE for the selective determination of 5-HT, different concentrations of 5-HT have been loaded on the surface of SPCE/ZCNT 0.1 NCs electrode and their respective electrochemical parameters have been analyzed using the SWV technique. The electrodes have been prepared as previously mentioned and 40 μL 5-HT in between 10 and 300 μM are dropped on SPCE/ZCNT 0.1 NCs electrodes. Figure 15 illustrates how the anodic peak at ~ 0.4 V versus Ag increases with increase in 5-HT content on the electrode surfaces and hence current response of this studies is varied linearly with concentrations of 5-HT. This trend is consistent with other electrochemical approaches for the detection of 5-HT within biological samples^17^.

**Figure 16:**
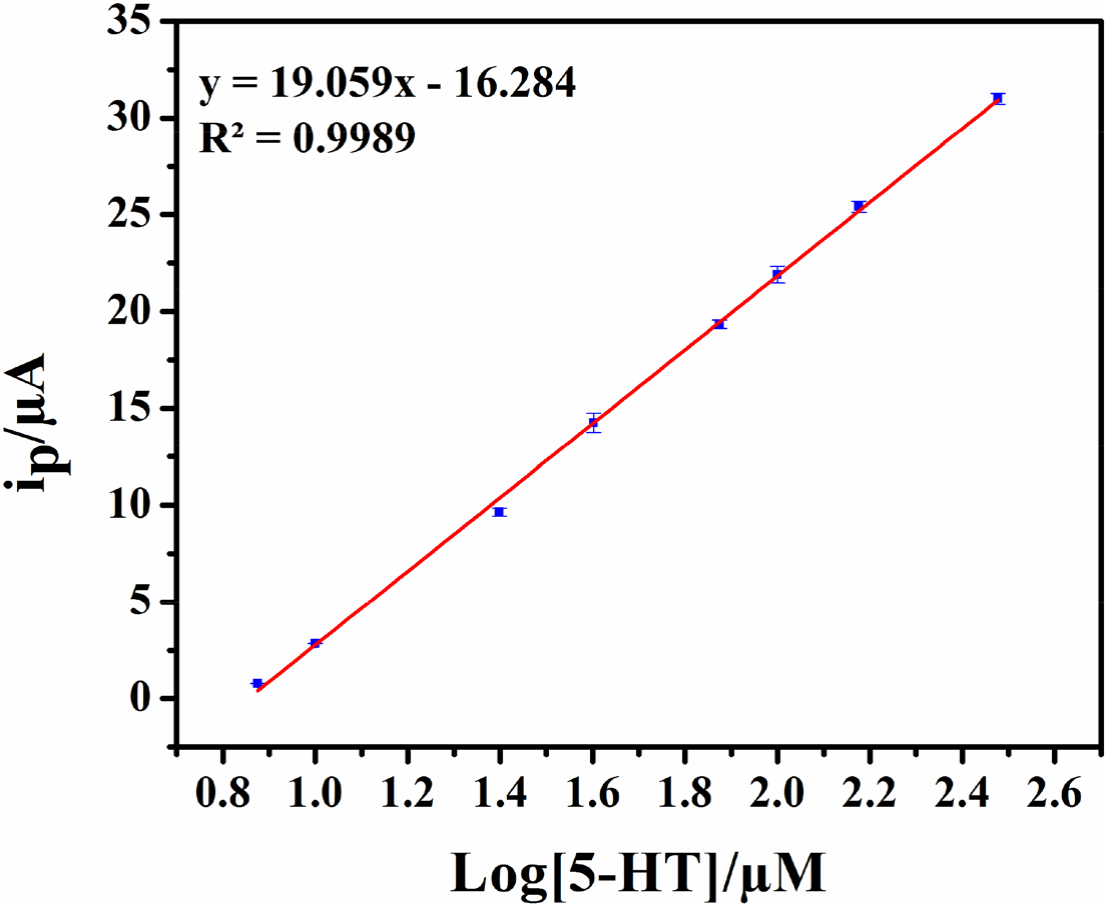
Linear range of the calibration curve for the detection of 5-HT performing SWV (Estep: 0.001 V; Amplitude: 0.05 V; Frequency: 25 Hz) on ZCNT 0.1 NCs modified SPCE. Error bars for n = 3 are included but are smaller than the point size.

When the data are plotted on a semi log scale (Fig. 16), a linear response is observed in the concentration range from 7.5 to 300 μM of 5-HT following the equation below.

$$\begin{aligned} & {\text{Y}} = {19}.0{59}\left[ {\text{5-HT}} \right] - {16}.{284} \\ & {\text{R}}^{{2}} = 0.{9989} \\ \end{aligned}$$

Using the standard deviation (SD) and obtained regression coefficient, the limit of detection (LOD) and the limit of quantification (LOQ) are calculated with the following equations:

$$\begin{aligned} & {\text{LOD}} = {\text{3SD}}/{\text{b}} \\ & {\text{LOQ}} = {1}0{\text{SD}}/{\text{b}} \\ \end{aligned}$$

where SD is the standard deviation and b is the slope of the analytical curve. The LOD and LOQ (S/N = 3) are calculated to be 0.66 μM and 2.19 μM respectively with wide linear range (7.5–300 μM) of analyte. Therefore, the LOD and linear range of present 5-HT sensing studies using SPCE/ZCNT 0.1 NCs is significantly good in comparison to the other reported materials^21,79–87^; which is shown in Table 3. As per our best knowledge, ease synthesized one dimensional (1D) (ZnO NRs)_1−x_(CNs)_x_ NCs have been reported first-time for efficient electrochemical 5-HT sensing studies. The efficient performance of this sensing protocol has been reflected through comparable lower LOD values (0.66 μM), wide range of detection range (7.5–300 μM) with less volume of sample required (40 µL) in comparable to other related investigations^88, 89^.

**Table 3:** Performance of previously reported modified electrodes for the detection of 5-HT.

Modified electrode	Linear region (μM)	Detection limit (μM)	References
Graphite conductive ink	6.0–100.0	0.39	^87^
5-HTP/GCE	5.0–35.0	1.7	^79^
Carbon fibre electrode	2.5–10.0	1.0	^80^
Au/OPPy	5.0–50.0	0.89	^86^
Poly(3,4 ethylenedioxythiophene) modified Pt electrode	20.0–100.0	71	^81^
IL/DC/f-CNT/GCE	5.0–900.0	2	^21^
f-MWCNTs/BR9	–	7	^82^
GNPs on Nafion/CPE	50.0–3,000.0	2.84	^83^
Nanohole array and GNPs	–	22.52	^84^
	–	10.61	
Tris(2-ethylhexyl)phosphine oxide membrane electrode	0.1–10,000	9	^85^
SPCE/ZCNT 0.1	7.5–300.0	0.66	Present work

*5-HTP* 5-hydroxytryptophan, *GCE* glassy carbon electrode, *CNT* carbon nanotube, *IL* ionic liquids, *ZCNT* zinc oxide-MWCNTs nanocomposite, *SPCE* screen printed carbon electrode, *Pt* platinum, *Au* gold, *OPPy* overoxidized polypyrrole, *f-MWCNTs/f-CNTs* functionalized multiwall carbon nanotubes, *BR9* basic red 9, *GNPs* gold nanoparticles, *CPE* carbon paste electrode.

### Reproducibility studies

**Figure 17:**
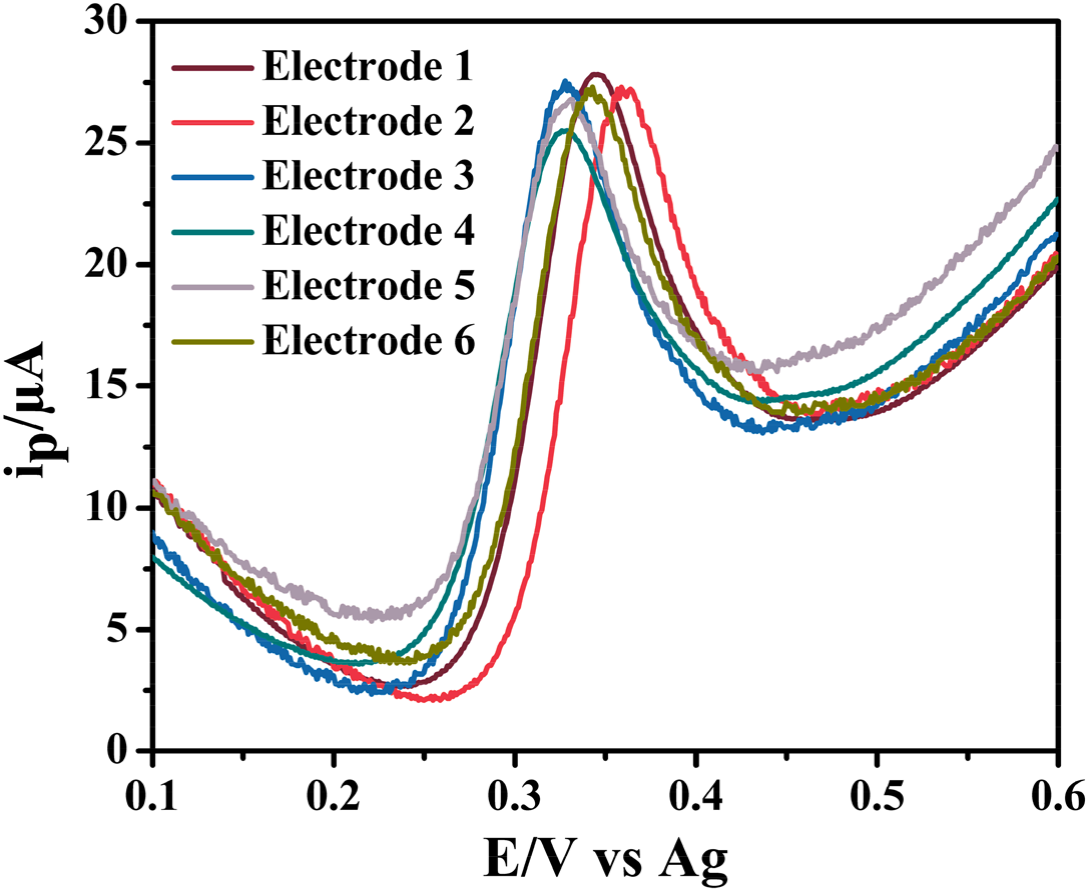
SWV response (Estep: 0.001 V; Amplitude: 0.05 V; Frequency: 25 Hz) from 6 different ZCNT 0.1 NCs modified SPCE on 100 μM 5-HT.

In addition, we have also conducted the reproducibility studies of SPCE/ZCNT 0.1 NCs for 5-HT sensing studies to illustrate the robustness of this approach. In this protocol, 40 μL of 100 μM 5-HT have been dropped on six different SPCE/ZCNT 0.1 NCs and thereafter their response has been tested through SWV analysis (shown in Fig. 17). In these six different sets of SWV analysis, the current response for 5-HT sensing studies is almost same and having an average of 17.5 ± 1.8 μA demonstrating the good reproducibility with an relative standard deviation of 10.2% over these six sets in the present sensing protocol. Therefore, potentially these materials can make a substantial impact on many analytical applications by offering a cheaper, more commercially viable electrode for portable sensing.

Common interferences include ascorbic acid (AA), uric acid (UA), and dopamine (DA). AA and UA show an oxidation peaks at ~ 0.05 V versus Ag/AgCl and so would be easily separated from the 5-HT peak^19^. We have previously shown that dopamine has a much higher oxidation potential^17,75^. Given this information, it is unlikely that these small biomolecules would interfere with the detection of 5-HT. This study highlights a proof-of-concept for the application of these novel materials as electrochemical sensing platforms. In future studies we will examine the full method development to illustrate their analytical applicability to this type of analysis.

## Conclusions

In the present investigation, we have synthesized one-dimensional (ZnO NRs)_1−x_(CNs)_x_ NCs through simple in-situ wet-chemical protocol and thereafter their structural properties as well as interconnectivity between the materials have been tested through spectroscopic (XRD, FTIR, Raman, XPS), microscopic (FESEM with EDS) and BET measurements. Afterthat, the (ZnO NRs)_1−x_(CNs)_x_ NCs have been successfully deposited on SPCE substrate using a drop casting technique for getting a well adherent and uniform electrode. Also, the electron transfer rate between the modified electrodes and electrolyte (ferrocene redox couple) is studied through EIS measurements. Among the different electrodes, ZCNT 0.1 NCs based electrode proved to be highly sensitive towards the oxidation of 5-HT and thereby it was systematically applied for the rest of electrochemical measurements. The unique beauty of this investigation is the development of efficient, sensitive 5-HT sensing protocol in terms of lower LOD values, wide detection range (7.5–300 μM) with less volume of sample (40 µL) in comparable to other related investigations. Although further work is needed to establish the utility of these material for detection within the appropriate concentrations ranges, and in real samples along with interference studies, the easy and quick preparation of this sensor make it suitable not only for 5-HT detection but also for other species of interest and for potential inclusion within portable platforms highlighting the potential of these novel materials as electrochemical detection platforms.

## Supplementary information

Supplementary Information.
