# Supplementary material for: Structural refinement and electrochemical properties of one dimensional (ZnO NRs)1−x(CNs)x functional hybrids for serotonin sensing studies
Source: Sci Rep. 2020 Sep 29;10:15955. doi: 10.1038/s41598-020-72756-3 (PMC7524834; doi:10.1038/s41598-020-72756-3)
Supplement: Supplementary file 1 — Supplementary Information. [file 41598_2020_72756_MOESM1_ESM.docx]

**Electronic Supporting Information (ESI)**

**Structural Refinement and Electrochemical properties of one-dimensional (ZnO NRs)_1-x_(CNs )_x_ functional hybrids for Serotonin Sensing Studies**

Sajid B. Mullani^a^, Ananta G. Dhodamani^a^,Annadanesh Shellikeri^b^, Navaj B. Mullani^c^, Anita K. Tawade^d^, Shivaji N. Tayade^a^, JulienBiscay^e^, Lynn Dennany^e^ and Sagar D. Delekar*^a^

^a^Department of Chemistry, Shivaji University, Kolhapur 416004 (MS), India

^b^ Department of Electrical and Computer Engineering, Florida A&M University-Florida State University, Tallahassee, FL 32310-6046, USA; and Aero-Propulsion, Mechatronics and Energy Centre, Florida State University, Tallahassee, FL 32310-6046, USA

^c^Department of Advanced Materials and Chemical Engineering, Hanyang University ERICA Ansan, 15588 South Korea

^d^School of Nanoscience and Biotechnology, Shivaji University Kolhapur 416004 (MS),India

^e^Department of Pure and Applied Chemistry, University of Strathclyde, Technology and Innovation Centre, 99 George Street, Glasgow, G1 1RD, U.K.

Submission of revised manuscript to

***Scientific Reports***

(Nature publications)

*Corresponding author:

Prof. (Dr.) Sagar D. Delekar

E-mail: [sddelekar7@rediffmail.com](mailto:sddelekar7@rediffmail.com)

(Tel.:+91 231 2609311, Fax: +91 231 2692333)

**1. Experimental Section**

**1.1 Materials**

Multi-walled carbon nanotubes (MWCNTs) [diameter: 20-45 nm, length: 10-40 µm and surface area: >500 m^2^/g] and Natural graphite powder [<20 µm, (synthetic)] were purchased from Sigma-Aldrich. Other chemicals are of AR-grade and used directly without purification.

**1.2 Functionalization of MWCNTs**

Commercially purchased pristine MWCNTs was functionalized through previously reported acid treatment methodwith slight modifications.[^1^](#_ENREF_1) Initially, calculated amount of raw MWCNTs powder transferd into the mixture of conc. H_2_SO_4_:HNO_3_ (3:1volume ratio) and refluxed at 90^°^C for 3 hrs. Then, the content was cooled at room temperature. The upper liquid portion was decanted and the remaining semi-solid portion was transferred into the double distilled water (DDW), then it was centrifuged, and againsemi-solid washed with DDW up to its neutralization and finally, dried in electric oven at 80 ^°^C.

**1.3 Synthesis of GO**

The oxidation of commercially purchased natural graphite powder was carried out through modified Hummers-Offemanmethod with slight modifications.[^2^](#_ENREF_2) Particularly, natural graphite powder and sodium nitrate (NaNO_3_) was mixed into the coldconc. H_2_SO_4_ (0^°^C) then the appropriate amount of grinded KMnO_4_ was slowly added into it with constant stirring and cooling. Afterthat, the whole mixture solution was kept in room temperature for 1 h. and then excess DDW was added into it; then temperature of the mixture was increases gently above 100 ^°^Cdue to the exothermic reaction with water and acid. Then, 30 % H_2_O_2_ solution was added into it; then brown colored precipitate was observed. Finally, precipitate was washed with DDWup to the pH of the solution becomes neutral and dried in electric oven at 80 ^°^Cup to dryness; the obtained brown powder indicates the oxidation of natural graphite into graphene oxide (GO).

**1.4 Synthesis of RGO**

Chemical reduction method was deployed for the reduction of GO to reduced GO (RGO). In which, the calculated amount of GO was dispersed in DDW through constant stirring and then subjected to ultrasonic treatment for 30 min. Then, sodium borohydrate (NaBH_4_) was slowly added into the above dispersed solution and whole mixture solution was refluxed at 90^°^C for 3 hrs., then brown colored solution becomes black indicates the excess functional moieties of the GO was reduced under reduction for forming the RGO. Then black precipitate solution was washed with DDW until the pH of the solution becomes neutral and dried in electric oven at 80 ^°^C to obtain the black colored RGO.

The CNs (MWCNTs and RGO) were confirmed through various spectral techniques and included in the supporting information (*SI*) as [XRD S1 (a and b), FTIR S2 (a and b), Raman S3 (a and b), XPS S4 and S5 and TEM S7 (a to f)].

2. Instrumentation of electrochemical study

To elucidate the suitability of (ZnO NRs)_1-x_(CNs)_x_ modified screen printed carbon electrode (SPCE) for the detection of 5-HT, electrochemical measurements were carried out using a CH instrument model 760D electrochemical analyzer. All experiments were carried out using a conventional three-electrode assembly, consisting of a modified SPCE working electrode, Pt-wire (counter electrode) and Ag (reference electrode). Cyclic voltammetry (CV) was carried out at a scan rate of 100 mV s^-1^and sample interval of 1 mV across a potential range outlined in each figure. For square wave voltammetry (SWV), a potential step of 0.001V with an amplitude of 0.050V and a frequency of 25 Hz in order to obtain the optimum waveform condition. All the measurements were made at room temperature.

**3. Results and Discussion**

**3.1 X-Ray diffraction analysis**

**
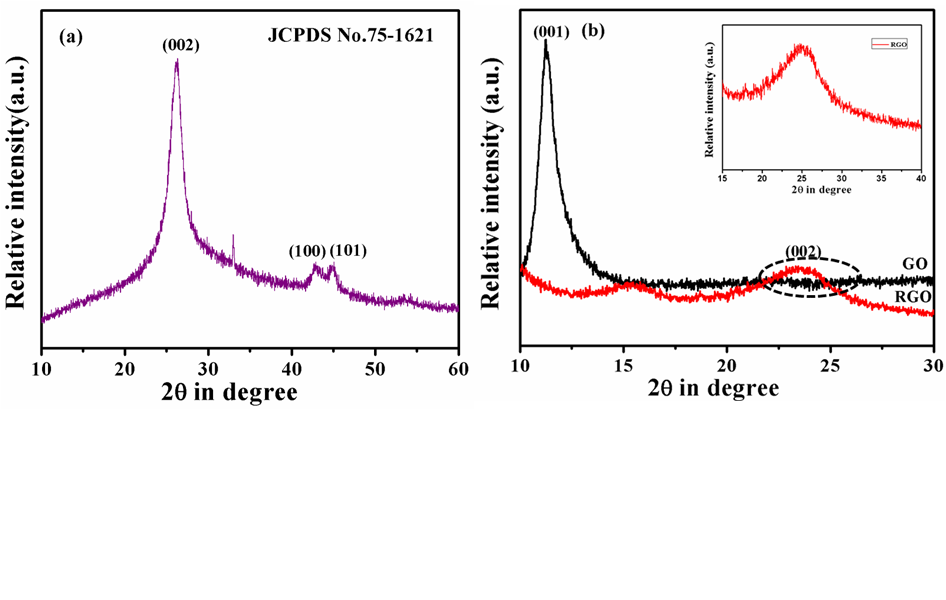
**

**Figure S1.**XRD diffractograms of (a) MWCNTs (b) GO & RGO.

The acid functionalized MWCNTs and functionalization of GO to RGO is confirmed through XRD analysis and displayed in figure S1 (a& b).MWCNTs indicate the characteristic graphitic carbon peak at 26.33˚, 42.87˚ and 44.98˚corresponding to (002), (100) and (101) reflections; respectively. All the peaks are clearly matched with the characteristic peaks of standard hexagonal graphitic carbon (JCPDS 75-1621).[^3^](#_ENREF_3)^,^ [^4^](#_ENREF_4)While, in case of GO After, chemical oxidation of GO depicts sharp diffraction peak at 2θ = 11.25˚(d-spacing = 0.7860 nm), which indicates that the graphite was fully oxidized into GO due to the insertion of oxygen containing functional groups between the layers.After the reduction of GO by using NaBH_4_, the peak at 11.25˚ disappeared and a broad peak appeared at 2θ =23.419˚(d-spacing = 0.3796 nm) [(enlarged XRD pattern;figureS1(b)]. The disappearance of the sharp peak can be attributed to the exfoliation of layered structures of GOor removal of oxygen containing functional groups, resulted in restacking of the RGO sheets and this observation confirms the GO converted to RGO.[^5^](#_ENREF_5)^,^ [^6^](#_ENREF_6)

**3.2 Relative XRD Intensity Ratio Analysis**

**
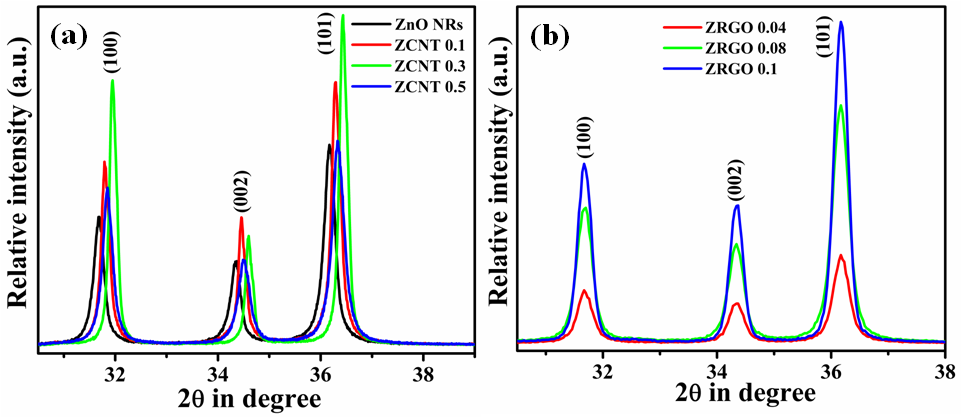
**

**Figure S2.** Relative XRD intensity ratio analysis of (a) bare ZnO NRs and (ZnO NRs)_1-x_(CNs)_x_ NCs with varying content of MWCNTs (0.1 to 0.5 wt.%) and (b) (ZnO NRs)_1-x_(CNs)_x_ NCs with varying content of RGO (0.04 to 1.0 wt.%) for (100),(002),(101) planes.

**Table S1** Relative XRD Intensity Ratio

XRD intensities (A.U.) and its relative intensities ratios (%) for the respectiveplanes

| **Composites** | **XRD intensities (A.U.) and its relative intensities ratios(%)** | | | | | |
| --- | --- | --- | --- | --- | --- | --- |
|  | **(100)** | **RIR (%)** | **(002)** | **RIR (%)** | **(101)** | **RIR (%)** |
| ZnO NRs | 5006.14 | 64.39 | 3287.64 | 42.28 | 7774.28 | 100 |
| ZCNT 0.1 | 7120.57 | 69.93 | 4986.32 | 48.97 | 10181.94 | 100 |
| ZCNT 0.3 | 10251.22 | 80.38 | 4269.84 | 33.57 | 12753.43 | 100 |
| ZCNT 0.5 | 6131.70 | 77.37 | 3354.93 | 42.33 | 7924.66 | 100 |
| ZRGO 0.04 | 1400.84 | 60.26 | 1051.90 | 45.25 | 2324.48 | 100 |
| ZRGO 0.08 | 3563.29 | 56.91 | 2613.06 | 41.73 | 6260.88 | 100 |
| ZRGO 0.1 | 4724.40 | 55.92 | 3626.40 | 42.92 | 8447.86 | 100 |

* A.U. –Arbitrary Unit, RIR – Relative Intensity Ratio

Powder XRD patterns were collected for identification of phases formed and to track the changes in the structure if any. Figure S2 displays the overlapping XRD patterns of bare ZnO NRs, ZCNT NCs [figure S2 (a)]and ZRGO NCs[figure S2 (b)]. Close inspection of XRD patterns overlapped indicates that the intensity of ZnO NRs refection is enhanced significantly after forming the composites with CNT upto ZCNT 0.3; NCs while that of ZCNT 0.5 NCs sample increases marginally than ZnO NRs. Apart from this the continuous shift towards higher values of 2θ position of the ZnO NRs reflections is observed up to ZCNT 0.3 NCs. This change in the pattern of ZCNT 0.5 NCs is not prominent which may highlight the limit of composition. The change in intensity and shift in 2θ values are in good harmony with each other. The shifts towards higher 2θ values indicate the successful incorporation of carbon from MWCNT in the ZnO NRs lattice (smaller atomic dimensions of carbon) which reduces its lattice size. In case of ZRGO NCs, intensity of XRD pattern reflections is enhanced significantly from ZRGO 0.04 to ZRGO 0.1 NCs; when reflections are compared with reflections of bare ZnO NRs, it indicates decrease in peak intensity up to ZRGO 0.08 NCs and increases marginally for ZRGO 0.1 NCs. The relative intensities ratio (RIR) of (100), (002) and (101) planes of ZCNT NCs are affected significantly while in case of ZRGO NCs; RIR values are only marginally affected.

**3.3 FTIR analysis**

Figure S3 (a&b) demonstrate the FTIR spectrum of MWCNTs and GO-RGO. The peak at 1029 cm^-1^corresponds toC–O stretching vibrations and 1506 cm^-1^corresponding to C=C stretching vibrations of nanotubes aromatic rings stretching in MWCNTs.[^7^](#_ENREF_7)^,^ [^8^](#_ENREF_8)Covalent functionalization of MWCNTs observed at 1726 cm^-1^ and it confirms the successfully functionalization of the oxygen moieties.[^9^](#_ENREF_9) The peak at 2881 cm^-1^assigned to C–H stretching vibration of methylene produced at the defect sites of acid-oxidized MWCNT surface.[^10^](#_ENREF_10)The FTIR spectrum of GO showing the many strong absorption peaks corresponding to various oxygen functional groups, such as, sharp carbonyl C=O (1725 cm^-1^), carboxyl O=C–O (1399 cm^-1^), epoxy C–O (1223 cm^-1^), alkoxy C–O (1054 cm^-1^) and aromatic C=C (1618 cm^-1^) stretching vibrations. The FTIR spectrum of RGO showed relatively weak stretching vibrations of C=O (1734 cm^-1^), aromatic C=C (1560 cm^-1^), and alkoxy C–O (1053cm^-1^). The O-containing functional groups are showing higher absorbance value in GO as compared to RGO.[^11^](#_ENREF_11) In MWCNTs, GO and RGO shows broad peak at 3400-3500 cm^-1^which is assigned to the O−H stretching. The spectrum of RGO shows no sharp peaks as compare GO to confirming the efficient reduction of GO to RGO.[^12^](#_ENREF_12)


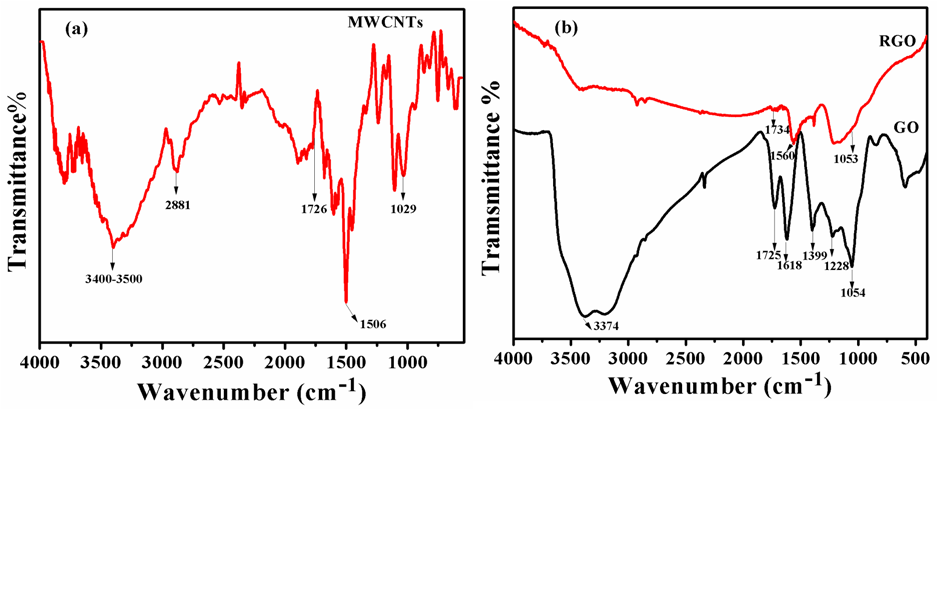


**Figure S3.** FTIR spectra of (a) MWCNTs (b) GO & RGO.

All observed data confirms the, attachments of strongly hydrogen-bonded hydroxyl moieties (O–H) and carbonyl group (C=O) to CNs, which correspond to the characteristic of carboxyl functional groups (–COOH).[^13^](#_ENREF_13)Here, functionalization of CNs is suitable for the chemical interconnection between ZnO NRs and CNs.

**3.4 Raman analysis**

**
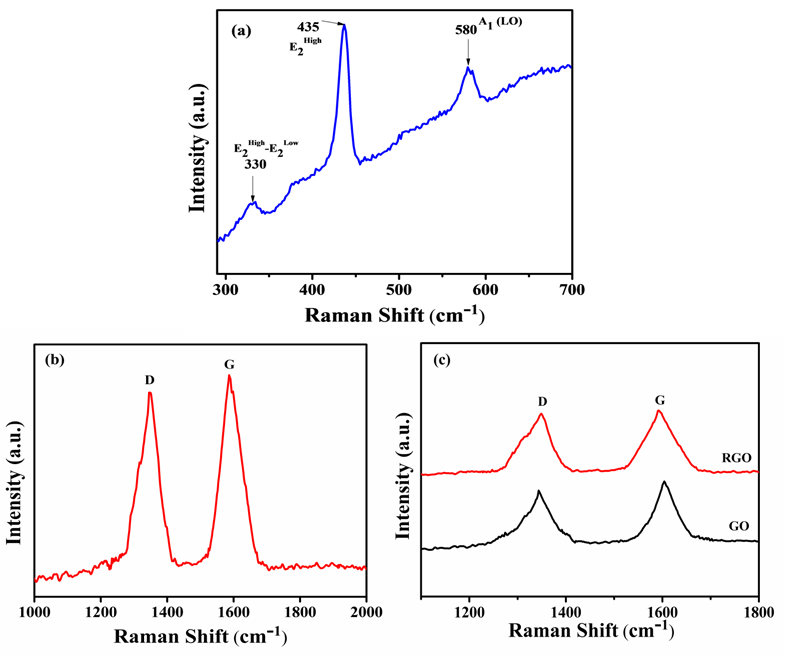
**

**FigureS4.** Raman spectra of (a) bare ZnO NRs (b) MWCNTs and (c)GO& RGO

Figure S4 (a) displays the Raman spectrum of ZnO NRs and it reveals the peaks at 580 cm^-1^, 435 cm^-1^and 330 cm^-1^ corresponding to the longitudinal optical A_1_(LO) mode due tostrucrural defects i.e. oxygen vacancies and Zn interstitials,E_2_^High^ (high frequency)due to the wurtzite phase of ZnO NRs, and the second order low-frequency E_2_ phonon mode E_2_ (Low) respectively.[^14^](#_ENREF_14)

Significant changes occurred in structures of CNs during the functionalization and thesestructural changes have been characterized by Raman analysis.FigureS4(b&c) consists of Raman spectrum for MWCNTs, GO & RGO. In MWCNTs,the peak of D band at ∼ 1345 cm^-1^due to the defects because of acid treatment, whereas the peak of G band at ∼ 1586 cm^-1^ also corresponds to the E_2g_ vibration mode corresponding to graphitic carbon.The ratio of intensity for D band to G band increases in functionalized MWCNTs (I_D_/I_G_=0.92) due to theincrease of defects as compared to pristine MWCNTs. This result shows the defects present on MWCNTs and it leads to structural deformations, without any change in graphite structure of MWCNTs surface. The intensity ratio of bands shows acidic functional moieties present on MWCNTs and it reveals that carbon atoms form sp^2^ to sp^3^ hybridization.[^15^](#_ENREF_15)[^16^](#_ENREF_16)The Raman spectrum of the GO shows the well-defined D band at 1343 cm^-1^ due to the sp^3^ defects and G band at 1604 cm^-1^ analogous to the E_2g_ phonon of sp^2^carbon which can be ascribed to the in-plane vibrations of sp^2^ carbon atoms and a doubly-degenerated phonon mode (E_2g_ symmetry) at the Brillouin zone centre. The Raman spectrum of RGO shows, there is no distinction in the D band while, G band is downshifted from 1604 cm^-1^ to1591cm^-1^owing to the “self-healing” characteristics of the RGO that recovers the hexagonal network of carbon atoms with defects it confirms the successful reduction of GO to RGO.[^17-19^](#_ENREF_17)In addition, the I_D_/I_G_ ratio was used to measure structural disorder which was slightly increased from 0.90 to 0.95 during the reduction of GO to RGO.[^20^](#_ENREF_20)As a result, the formations of defects occur and the removal of oxygen functional groups attached from GO surface.[^21^](#_ENREF_21)[^22^](#_ENREF_22)

**3.5 XPS analysis**

The figure S5 shows the survey spectrum and core level spectrums of chemically acid modified multiwalledcarbon nanotube. Spectra depicts the high-resolution and curve fitting of C1s [figure S5 (b)]and O1s [figure S5 (c)]spectrums of acid functionalized MWCNTs. As expected, the amount of oxygen increases after acid treatment.

**
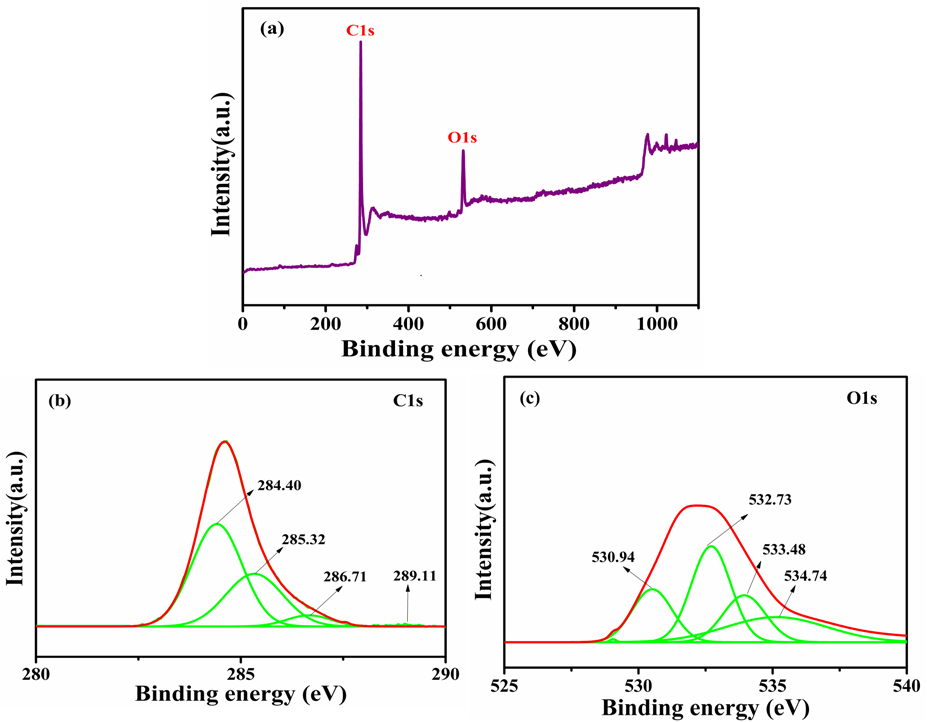
**

**FigureS5.** (a) XPS survey spectrum and high resolution core level XPS spectrum of (b) C1s and (c) O 1s of the representative MWCNTs.

The majorpeak assigned at 284.40 eV represents the C1s binding energy of typical graphitic carbon peak. Furthermore, the O1s peak is significantly broader in the MWCNTs and these characteristics peaks such as 530.94 eV, 532.73 eV, 533.48 eV and 534.74 eV can be associated to carbon and oxygen interactions such as hydroxyl(–OH), carbonyl (C=O), and carboxyl groups(–COOH). Among the all peaks; high intense peak observed at 532.73 eV can be assigned to oxygen species in carboxylic group (C=O).[^16^](#_ENREF_16)^,^ [^23^](#_ENREF_23) While, C1s was deconvoluted into four binding energies at 284.40, 285.32, 286.71 and 289.11 corresponding to C–C, C–O, C=O and COO^–^species respectively.[^24^](#_ENREF_24)^,^ [^25^](#_ENREF_25)These results provide additional evidence that oxygen-based functional groups were introduced to the multiwalled nanotube surface.

**
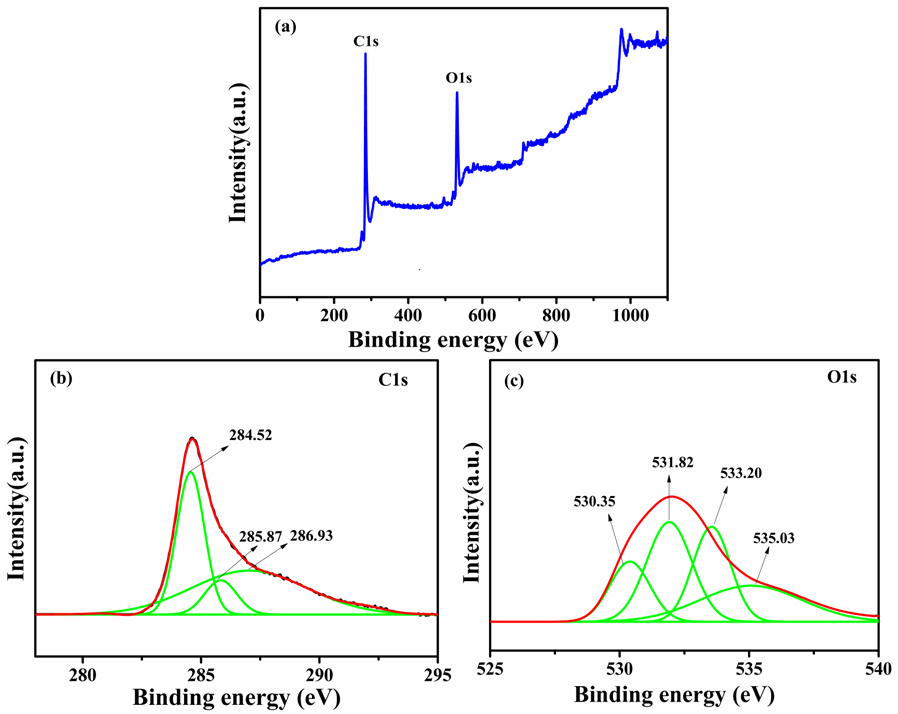
**

**FigureS6.** (a) XPS survey spectrum and high-resolution core level XPS spectrums of (b) C1s and (c) O1s of the representative RGO.

The figure S6shows the survey spectrum and core level spectrums of GO to RGO functionalization.The XPS spectra were analyzed to determine the surface functional groups of RGO. The existence of residue oxygen groups and carbon was demonstrated by C1s[figure S6 (b)] and O1s[figure S6 (c)] XPS core level spectrums. Asymmetrical C1s binding energy bands were observed in pure RGO and further deconvoluted into three components and it observed at 284.52eV due to graphitic carbon, 285.87eVdue to carbon atoms with sp^3^ hybridization,286.93eVdue to C–OH. After reduction, the C–C group occupied the maximum surface and it revealed that oxygen containing functional groups were mostly removed and the graphene network keeps constant. The O1s spectra deconvoluted into four peaks described as quinone typeC(O)O (530.35), C–OH (531.82), –C–O (533.20) and chemisorbed oxygen (carboxylic groups) and/or water (535.03). These all functional moieties confirm the GO converted to RGO.[^11^](#_ENREF_11)^,^ [^26^](#_ENREF_26)

**
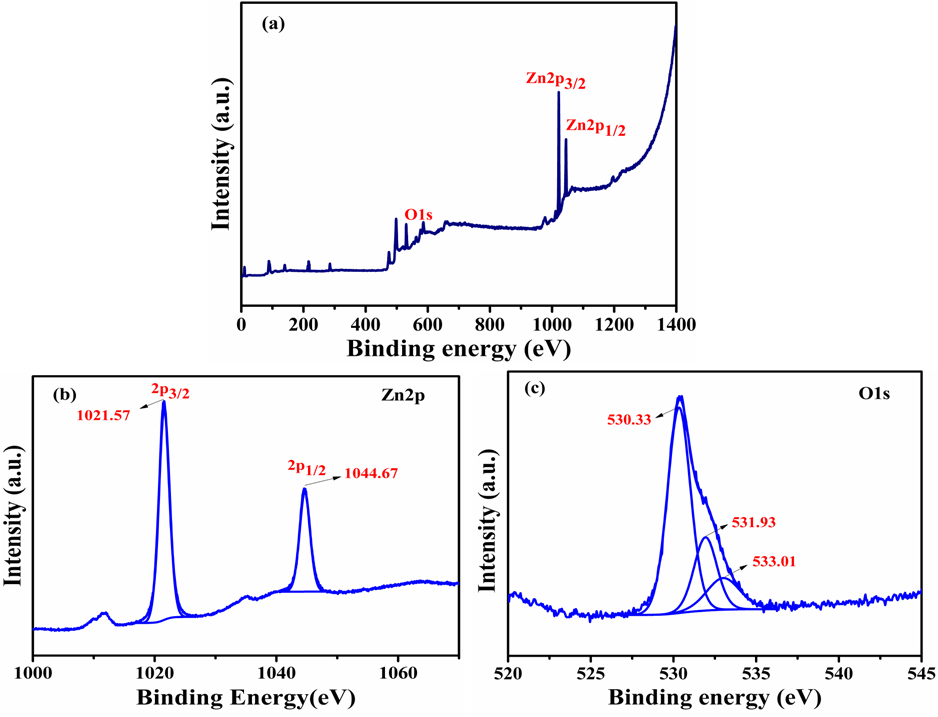
**

**Figure S7.**(a) XPS survey spectrum and high resolution core level XPS spectrums of (b) Zn 2p and (c) O 1s of the representative bare ZnO NRs.

Figure S7 (a) shows that full XPS spectrum of Zn and O elements. The high resolution XPS spectrum of Zn2p shown in figureS7 (b) clearly indicates that the two peaks positioned at 1021.57 eV and 1044.67 eV corresponding to Zn 2p_3/2_ and Zn 2p_1/2_, respectively. The energy difference between these two peaks is 23.1eV, clearly illustrating that zinc species is in the formal Zn^2+^ valence state.[^27^](#_ENREF_27)^,^ [^28^](#_ENREF_28) The figureS7 (c) shows, the O1s peak of ZnO NRs can be deconvoluted into three spectral components, respectively, centered at 533.01, 531.93, and 530.33 eV. Out of three, the component with lowest binding energy O 1s peaks at 530.33 eV assigned to lattice oxygen in ZnO. The one with the medium binding energy centered at 531.93 eV corresponds to the O^2−^ ions in the O deficient regions & it called deficient oxygen, and the highest binding energy centered at 533.01 corresponds to the chemisorbed O species on the surface of ZnO, such as H_2_O.[^29^](#_ENREF_29)

**3.6 TEM analysis**


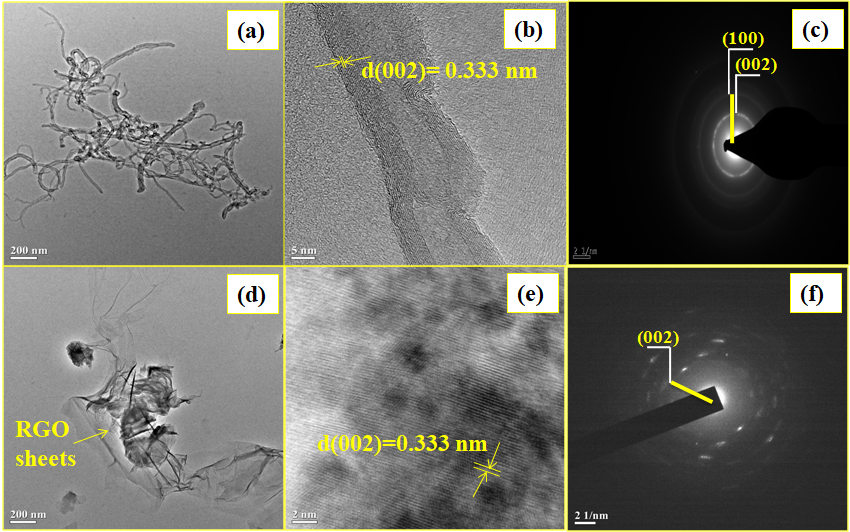


**FigureS8.**TEM-HRTEM with SAED patterns of MWCNTs (a, b, c) and RGO (d, e, f).

The straight evidence of the formation of acid functionalizedMWCNTs and functionalization of GO to RGO shows given by TEM, HR-TEM and SAED pattern in figure S8. TEM image of MWCNTs is shown in figureS8 (a) which exhibits some direct visualization of the length, diameter distribution and the defects of MWCNT after acid treatment. The more individual CNTs appear after treated with H_2_SO_4_:HNO_3_ (3:1), indicating the use of mixed acid is an effective way to cut the highly twisted long ropes of CNTs into short, open-ended pipes. Moreover, high-resolution TEM image showed rough surfaces of MWCNTs which might reveal that new defects were generated on the surface of the carbon nanotube because of the covalent functionalization. These defects provide a reactive site to the reagent and thus binding with oxygenated species strongly.FigureS8 (d) shows the TEM image of RGO showed that the single or few layers of RGO nanosheets were not perfectly flat and it showed the presence of wrinkles ripples and scrolls in the RGO suggesting the existence of few-layered graphene sheets. The HR-TEM of MWCNTs and RGO figureS8 (b, e) shows the width of fringes are the same (0.333 nm) and it is approximately matched with calculated d-spacing value of XRD analysis.

Furthermore, SAED patterns of both the CNs figureS8 (c,f) illustrates the formation of crystalline states of CNs. Distinct, bright ring pattern in SAED patterns confirms the formation of nanocrystalline material. The indexing values of CNs are closely matched with calculated values from XRD patterns.

**Table S2.** Thermogravimetric Analysis

Thermal studies of ‘as-prepared’ zinc hydroxide precursor with or without CNs [RGO= 0.04 wt % and MWCNTs= 0.1 wt% ]

| **Temp. Range**  **(^°^C)** | **Weight Loss (%)** | | | **Loss of chemical moieties** |
| --- | --- | --- | --- | --- |
|  | **ZnO**  **NRs** | **ZRGO0.04 NCs** | **ZCNT0.1**  **NCs** |  |
| RT to 125 | 2.75 | 6.97 | 9.33 | Lattice water or water of crystallization |
| 125 to 175 | 4.96 | 5.12 | 10.35 | Co-ordinated water |
| 175 to 400 | 5.98 | 22.18 | 17.79 | Organic moieties from PEG, Zn-precursor and combustion of CNs |
| Beyond 400 | Negligible | Negligible | Negligible | _ |

**Table S3.** XRD parameters

Observed structural cell parameters (XRD profile) of ZnO NRs and (ZnO NRs)_1-x_(CNs)_x_ NCs with varying content of CNs (MWCNTs =0.1 to 0.5 wt % and RGO= 0.04 to 0.1 wt % )

| **Composites** | **Standard**  **dvalues**  **(Å)** | **Observed d values**  **(Å)** | **(hkl)**  **plane** | **Cell parameters** | | | **Crystallite size**  **(nm)** |
| --- | --- | --- | --- | --- | --- | --- | --- |
|  |  |  |  | **a (Å)** | **c (Å)** | **V(Å^3^)** |  |
| **ZnO NRs** | 2.6033 | 2.6085 | (002) | 3.2532 | 5.2169 | 55.1035 | 19 |
|  | 2.4759 | 2.4824 | (101) |  |  |  |  |
|  | 1.6247 | 1.6266 | (110) |  |  |  |  |
| **ZCNT 0.1** | 2.6033 | 2.6014 | (002) | 3.2532 | 5.2028 | 55.0628 | 23 |
|  | 2.4759 | 2.4736 | (101) |  |  |  |  |
|  | 1.6247 | 1.6266 | (110) |  |  |  |  |
| **ZCNT 0.3** | 2.6033 | 2.5914 | (002) | 3.2408 | 5.1828 | 54.4338 | 25 |
|  | 2.4759 | 2.4645 | (101) |  |  |  |  |
|  | 1.6247 | 1.6204 | (110) |  |  |  |  |
| **ZCNT 0.5** | 2.6033 | 2.5992 | (002) | 3.2454 | 5.1984 | 54.7527 | 18 |
|  | 2.4759 | 2.4716 | (101) |  |  |  |  |
|  | 1.6247 | 1.6227 | (110) |  |  |  |  |
| **ZRGO 0.04** | 2.6033 | 2.6089 | (002) | 3.2532 | 5.2178 | 55.2215 | 25 |
|  | 2.4759 | 2.4816 | (101) |  |  |  |  |
|  | 1.6247 | 1.6266 | (110) |  |  |  |  |
| **ZRGO 0.08** | 2.6033 | 2.6089 | (002) | 3.2518 | 5.2178 | 55.1740 | 26 |
|  | 2.4759 | 2.4824 | (101) |  |  |  |  |
|  | 1.6247 | 1.6259 | (110) |  |  |  |  |
| **ZRGO 0.1** | 2.6033 | 2.6080 | (002) | 3.2550 | 5.2160 | 55.2636 | 29 |
|  | 2.4759 | 2.4824 | (101) |  |  |  |  |
|  | 1.6247 | 1.6275 | (110) |  |  |  |  |

**Table S4.**Experimetally Observed Raman peakpositions and intensity ratio for D-band and G-bandof CNs in the bare or NCs.

| **Composites** | **Raman D-band**  **(cm^-1^)** | **Raman G-band**  **(cm^-1^)** | **Ratio**  **I_D_/I_G_** |
| --- | --- | --- | --- |
| **MWCNTs** | 1345 | 1586 | 0.92 |
| **RGO** | 1343 | 1591 | 0.95 |
| **ZCNT 0.1** | 1343 | 1580 | 0.98 |
| **ZRGO 0.04** | 1362 | 1596 | 0.92 |

**Table S5.**EDS Composition

Theoretical and experimental elemental composition of ZnO NRs and (ZnO NRs)_1-x_(CNs)_x_ NCs with varying content of CNs (MWCNTs =0.1, 0.5 wt % and RGO= 0.04,0.1 wt % )

| **Composites** | **Theoretical** | | | **Experimental** | | |
| --- | --- | --- | --- | --- | --- | --- |
| **Elements** | **Zn** | **O** | **C** | **Zn** | **O** | **C** |
| **ZnO NRs** | 80.34 | 19.66 | – | 79.43 | 18.19 | – |
| **ZCNT 0.1** | 80.26 | 19.63 | 0.09 | 78.47 | 18.17 | – |
| **ZRGO 0.04** | 80.29 | 19.67 | 0.03702 | 78.25 | 19.21 | 2.54 |

**Table S6.**BET Analysis

Specific surface, pore volume and pore size from BET analysis of representative ZnO NRs and ZCNT 0.1 and ZRGO 0.04 NCs

| **Composites** | **Specific surface Area (m^2^/g)** | **Pore volume**  **(cm^3^/g)** | **Pore size**  **(nm)** |
| --- | --- | --- | --- |
| **ZnO NRs** | 16.699 | 0.530 | 3.820 |
| **ZCNT 0.1** | 97.895 | 0.614 | 17.417 |
| **ZRGO 0.04** | 55.078 | 0.345 | 3.409 |

All electrochemical data underpinning the detection of 5-HT in this publication are openly available from the University of Strathclyde Knowledge Base at following link. <https://doi.org/10.15129/d0a22009-122a-4bbc-8f4b-9a4197cd2ee6>

**Electronic Supporting Information References**

1. Patel, A.K., Trivedi, P. & Balani, K. Carbon nanotube functionalization decreases osteogenic differentiation in aluminum oxide reinforced ultrahigh molecular weight polyethylene. *ACS Biomater. Sci. Eng.* **2**, 1242-1256 (2016).

2. Wu, X., Xing, Y., Pierce, D. & Zhao, J.X. One-Pot Synthesis of Reduced Graphene Oxide/Metal (Oxide) Composites. *ACS Appl. Mater. Interfaces* **9**, 37962-37971 (2017).

3. Koli, V.B., Dhodamani, A.G., Delekar, S.D. & Pawar, S.H. In situ sol-gel synthesis of anatase TiO2-MWCNTs nanocomposites and their photocatalytic applications. *"J. Photochem. Photobiol., A"* **333**, 40-48 (2017).

4. Xia, X.*, et al.* A new type of porous graphite foams and their integrated composites with oxide/polymer core/shell nanowires for supercapacitors: structural design, fabrication, and full supercapacitor demonstrations. *Nano Lett.* **14**, 1651-1658 (2014).

5. Sookhakian, M.*, et al.* Enhanced photovoltaic performance of polymer hybrid nanostructure heterojunction solar cells based on poly (3-hexylthiophene)/ZnS/ZnO/reduced graphene oxide shell–core nanorod arrays. *Industrial & Engineering Chemistry Research* **53**, 14301-14309 (2014).

6. Mishra, S.K., Tripathi, S.N., Choudhary, V. & Gupta, B.D. SPR based fibre optic ammonia gas sensor utilizing nanocomposite film of PMMA/reduced graphene oxide prepared by in situ polymerization. *"Sens. Actuators, B"* **199**, 190-200 (2014).

7. Vinayan, B.*, et al.* Synthesis of graphene-multiwalled carbon nanotubes hybrid nanostructure by strengthened electrostatic interaction and its lithium ion battery application. *J. Mater. Chem.* **22**, 9949-9956 (2012).

8. Das, R., Hamid, S.B.A. & Annuar, M.S.M. Highly efficient and stable novel nanobiohybrid catalyst to avert 3, 4-dihydroxybenzoic acid pollutant in water. *Sci. Rep.* **6**, 33572 (2016).

9. Kim, B. & Sigmund, W.M. Functionalized multiwall carbon nanotube/gold nanoparticle composites. *Langmuir* **20**, 8239-8242 (2004).

10. Singh, B.P.*, et al.* Solvent free, efficient, industrially viable, fast dispersion process based amine modified MWCNT reinforced epoxy composites of superior mechanical properties. *Adv Mater Lett* **6**, 104-113 (2015).

11. Chen, X. & Chen, B. Macroscopic and spectroscopic investigations of the adsorption of nitroaromatic compounds on graphene oxide, reduced graphene oxide, and graphene nanosheets. *Environ. Sci. Technol.* **49**, 6181-6189 (2015).

12. Emiru, T.F. & Ayele, D.W. Controlled synthesis, characterization and reduction of graphene oxide: A convenient method for large scale production. *Egyptian Journal of Basic and Applied Sciences* **4**, 74-79 (2017).

13. Yee, M.J., Mubarak, N.M., Khalid, M., Abdullah, E.C. & Jagadish, P. Synthesis of polyvinyl alcohol (PVA) infiltrated MWCNTs buckypaper for strain sensing application. *Sci. Rep.* **8**, 17295 (2018).

14. Senthilkumar, N.*, et al.* Synthesis of ZnO nanoparticles using leaf extract of Tectona grandis (L.) and their anti-bacterial, anti-arthritic, anti-oxidant and in vitro cytotoxicity activities. *New J. Chem.* **41**, 10347-10356 (2017).

15. Nayak, P., Anbarasan, B. & Ramaprabhu, S. Fabrication of Organophosphorus Biosensor Using ZnO Nanoparticle-Decorated Carbon Nanotube–Graphene Hybrid Composite Prepared by a Novel Green Technique. *The Journal of Physical Chemistry C* **117**, 13202-13209 (2013).

16. Duc Chinh, V.*, et al.* Synthesis of Gold Nanoparticles Decorated with Multiwalled Carbon Nanotubes (Au-MWCNTs) via Cysteaminium Chloride Functionalization. *Sci. Rep.* **9**, 5667 (2019).

17. Basu, A.K., Sah, A.N., Pradhan, A. & Bhattacharya, S. Poly-L-Lysine functionalised MWCNT-rGO nanosheets based 3-d hybrid structure for femtomolar level cholesterol detection using cantilever based sensing platform. *Sci. Rep.* **9**, 3686 (2019).

18. Tatykayev, B.*, et al.* Synthesis of Core/Shell ZnO/rGO Nanoparticles by Calcination of ZIF-8/rGO Composites and Their Photocatalytic Activity. *ACS Omega* **2**, 4946-4954 (2017).

19. Zheng, Q.*, et al.* Highly transparent and conducting ultralarge graphene oxide/single-walled carbon nanotube hybrid films produced by Langmuir–Blodgett assembly. *J. Mater. Chem.* **22**, 25072-25082 (2012).

20. Guex, L.G.*, et al.* Experimental review: chemical reduction of graphene oxide (GO) to reduced graphene oxide (rGO) by aqueous chemistry. *Nanoscale* **9**, 9562-9571 (2017).

21. Aunkor, M.T.H., Mahbubul, I.M., Saidur, R. & Metselaar, H.S.C. The green reduction of graphene oxide. *RSC Adv.* **6**, 27807-27828 (2016).

22. Ding, J.*, et al.* Hydrothermal synthesis of zinc oxide-reduced graphene oxide nanocomposites for an electrochemical hydrazine sensor. *RSC Adv.* **5**, 22935-22942 (2015).

23. Gong, H., Kim, S.-T., Lee, J.D. & Yim, S. Simple quantification of surface carboxylic acids on chemically oxidized multi-walled carbon nanotubes. *Appl. Surf. Sci.* **266**, 219-224 (2013).

24. Yan, X.-b., Tay, B.K. & Yang, Y. Dispersing and functionalizing multiwalled carbon nanotubes in TiO2 sol. *J. Phys. Chem. B* **110**, 25844-25849 (2006).

25. Sun, H.*, et al.* Fabrication of polyimide and functionalized multi-walled carbon nanotubes mixed matrix membranes by in-situ polymerization for CO2 separation. *Sep. Purif. Technol.* **177**, 327-336 (2017).

26. Mohandoss, M., Gupta, S.S., Nelleri, A., Pradeep, T. & Maliyekkal, S.M. Solar mediated reduction of graphene oxide. *RSC Adv.* **7**, 957-963 (2017).

27. Wang, T.*, et al.* Photo-directed growth of Au nanowires on ZnO arrays for enhancing photoelectrochemical performances. *J. Mater. Chem. A* **2**, 15553-15559 (2014).

28. Ding, J.*, et al.* Synthesis of ZnO–Ag hybrids and their gas-sensing performance toward ethanol. *Industrial & Engineering Chemistry Research* **54**, 8947-8953 (2015).

29. Ahmad, R.*, et al.* Highly Efficient Non-Enzymatic Glucose Sensor Based on CuO Modified Vertically-Grown ZnO Nanorods on Electrode. *Sci. Rep.* **7**, 5715 (2017).
